# Ensemble method for dengue prediction

**DOI:** 10.1371/journal.pone.0189988

**Published:** 2018-01-03

**Authors:** Anna L. Buczak, Benjamin Baugher, Linda J. Moniz, Thomas Bagley, Steven M. Babin, Erhan Guven

**Affiliations:** 1 Johns Hopkins University Applied Physics Laboratory, Laurel, Maryland, United States of America; 2 Duke University, Durham, North Carolina, United States of America; University of Massachusetts, UNITED STATES

## Abstract

**Background:**

In the 2015 NOAA Dengue Challenge, participants made three dengue target predictions for two locations (Iquitos, Peru, and San Juan, Puerto Rico) during four dengue seasons: 1) peak height (i.e., maximum weekly number of cases during a transmission season; 2) peak week (i.e., week in which the maximum weekly number of cases occurred); and 3) total number of cases reported during a transmission season. A dengue transmission season is the 12-month period commencing with the location-specific, historical week with the lowest number of cases. At the beginning of the Dengue Challenge, participants were provided with the same input data for developing the models, with the prediction testing data provided at a later date.

**Methods:**

Our approach used ensemble models created by combining three disparate types of component models: 1) two-dimensional Method of Analogues models incorporating both dengue and climate data; 2) additive seasonal Holt-Winters models with and without wavelet smoothing; and 3) simple historical models. Of the individual component models created, those with the best performance on the prior four years of data were incorporated into the ensemble models. There were separate ensembles for predicting each of the three targets at each of the two locations.

**Principal findings:**

Our ensemble models scored higher for peak height and total dengue case counts reported in a transmission season for Iquitos than all other models submitted to the Dengue Challenge. However, the ensemble models did not do nearly as well when predicting the peak week.

**Conclusions:**

The Dengue Challenge organizers scored the dengue predictions of the Challenge participant groups. Our ensemble approach was the best in predicting the total number of dengue cases reported for transmission season and peak height for Iquitos, Peru.

## Introduction

Dengue fever is a leading cause of illness in tropical and subtropical regions of the world [1]. This disease is caused by a virus that has at least four different serotypes, so that previous infection with one serotype does not protect the individual from infection with the other three. Indeed, if the same person becomes re-infected with a different serotype, they have an increased risk of developing more serious symptoms, including dengue hemorrhagic fever and dengue shock syndrome [2]. The virus can be carried by *Aedes aegypti* or *Aedes albopictus* mosquitoes, which transmit the virus from person to person. A detailed review of how mosquitoes become competent vectors for disease transmission may be found in Kramer and Ciota [3]. Symptoms typically appear 4–7 days after an infected mosquito bites the individual. Symptoms range from extremely mild to severe, and may include fever as high as 41 C, headaches, post-ocular pain, myalgia, and arthralgia. Some patients also develop a widespread skin rash, nausea, and vomiting. Most patients recover within about one week. However, some patients become worse and develop severe abdominal pain, persistent vomiting, hemorrhage (gums, nose, under the skin, gastrointestinal), and complications involving the heart, liver, and lungs [1]. Currently, treatment is supportive and vaccines continue under development. Although dengue is endemic in at least 100 countries in the tropics and subtropics, these areas periodically experience increased risk when mosquito populations increase in proximity to susceptible human habitation. Environmental factors are known to influence the prevalence of mosquito vectors and risks of humans becoming infected with dengue [e.g., 4–6]. Because of the possibility of severe symptoms and death, there is great interest in predicting these disease outbreaks [6–8].

Previously, most models developed to predict future disease occurrences have relied upon different types of time series regression. These time series regression approaches assumed that future values could be predicted based upon past observations and were based upon retrospective determination of statistically significant correlation coefficients. However, as noted in the review by Nsoesie et al. [9], such correlation coefficients more accurately measure data trends instead of how close a prediction is to the measured data. Furthermore, these approaches often suffer from autocorrelation artifacts [10, 11] and so far, have not often been successfully used to predict a specific disease for times or locations not used in model development [12,13]. However, there are now many other approaches to modeling that seek to overcome these limitations. For example, noting a lack of predictive validity among previous studies, Lowe et al. [12] used a spatio-temporal generalized linear mixed model with parameters estimated in a Bayesian framework, and provided probabilistic early warnings for pre-defined alert thresholds for dengue, which was validated using out of sample predictions. Chen et al. [13] noted that many epidemic forecasting models fit existing data but fail to offer accurate predictions, so they used a reversed moving approximate entropy algorithm and pattern recognition on a time series of weekly dengue cases.

More recently, ensemble methods using two or more models with synthesized outputs have been employed. As in weather forecasting, the premise of the ensemble approach is that employing numerous models or many different variations of a similar model provides better accuracy than any single model. Because different single models often work well in certain situations but not in others, an ensemble approach may provide better results than any of its component models (i.e., the whole may be better than any of its parts) [14]. Ensemble modeling is now being used in a variety of disciplines, including disease prediction [e.g., 15,16]. Loshini et al. [16] found that ensemble models have better predictive power than single models when dengue incidence in a district was estimated using a dengue prediction model of that district together with its neighboring districts. Shaman et al. [17] used an ensemble prediction model for influenza and compared their ensemble forecasts of influenza outbreak peak timing with an alternate Method of Analogues approach similar to that used in numerical weather forecasting. Morin et al [18] used an ensemble approach to couple a mosquito life-cycle model with a susceptible-exposed-infectious-recovered model for dengue outbreaks in San Juan, Puerto Rico. Yamana et al. [19] separated training from testing data and used Bayesian averaging methods to combine three different ensemble prediction systems into a superensemble for forecasting dengue in San Juan, Puerto Rico.

Due to public health concerns about the spread and increasing extent of dengue, the US Centers for Disease Prediction and Control, the National Oceanic and Atmospheric Administration (NOAA), the US Department of Defense, the US Department of Health and Human Services, the US Department of Homeland Security, and other US government agencies joined together to sponsor a Dengue Forecasting Challenge project in 2015 [20]. The focus of the project was on predicting key metrics for historical dengue seasons using only data from time periods prior to those seasons. The participants were given identical historical data at the beginning of the Challenge, with the prediction testing data provided at a later date. Beginning on 5 June 2015, participants were provided with historical dengue surveillance data on which to develop and test the capabilities of their models. The dengue data were from Iquitos, Peru, and San Juan, Puerto Rico. In addition, historical environmental data from NOAA were provided along with relevant metadata. Participants were allowed to use other data sources such as social media or demographic data, but these data had to be readily available to all participants and approved by the contest organizers. The above data were to be used by the modelers to make the following types of predictions for a given dengue transmission season (a 12-month period commencing with the location-specific, historical week with lowest number of dengue cases):

1. Peak height—the number of dengue cases reported during the week when the number of cases reaches the maximum in a given dengue transmission season (also called peak incidence).
2. Peak week—the week when the highest number of weekly cases of dengue (i.e., peak height) occurs during the dengue transmission season.
3. Total number of cases in a transmission season—the number of dengue cases reported in the dengue transmission season (also called seasonal incidence).

The above target definitions come from the NOAA Dengue Challenge, although weekly incidence was used interchangeably with the number of dengue cases reported during the week. So, dengue incidence as described by the Challenge is not the number of dengue cases divided by population size but just the number of dengue cases. We will use the term incidence in the same way it was used by the Challenge organizers [20].

On 2 September 2015, final model submissions were submitted for review by an evaluation team led by representatives of the US Department of Health and Human Services. Finalists were invited to a meeting with representatives of the US National Science and Technology Council’s (NSTC) Interagency Pandemic Prediction and Forecasting Science and Technology Working Group at the Executive Office of the President in the fall of 2015. The purpose of this meeting was for participants to describe their models and provide their viewpoints on lessons learned and potential next steps in strengthening infectious disease forecasting, consistent with the US Federal Advisory Committee Act. This paper describes the approach used by one of the winning teams of this event. We apply an ensemble model for making the above dengue predictions for San Juan, Puerto Rico, and Iquitos, Peru.

## Materials and methods

### Data set description

#### Dengue data (Iquitos and San Juan)

Historical dengue surveillance data for San Juan, Puerto Rico, and Iquitos, Peru, were posted on the NOAA Challenge website [20]. For both locations, these weekly data included laboratory-confirmed dengue cases for each of the four serotypes plus laboratory-confirmed cases without serotype identification (acute IgM positive and IgM conversions). For Iquitos, the total dengue cases were the weekly sum of the lab-confirmed cases both specified and unspecified by serotype. For San Juan, however, the Challenge data also included additional cases where not all specimens were tested due to overload of the capacity for testing and incomplete case information. For these additional San Juan cases, the Challenge organizers provided weekly dengue case numbers derived by multiplying the number of untested cases by the rate of lab-positive cases among those that were tested. The total cases for San Juan were the weekly sum of the lab-confirmed cases both specified and unspecified by serotype plus the additional cases derived from estimates of untested specimens. Therefore, it is important to note that the San Juan data included dengue cases based on the assumption that the untested specimens would have the same percentage of positives for dengue as the tested specimens.

For both San Juan and Iquitos, these total weekly dengue cases were used for training and as prediction targets. In addition, these data were retroactively adjusted so that the case counts for each week reflected final weekly counts, whether or not the complete data were available at the time of the report [20]. Data were obtained in two installments: data on which to train models (for Iquitos, 2000–2009; for San Juan, 1990–2009) were posted at the beginning of the Challenge, while the prediction test data (remainder of 2009 through 2013) were posted several weeks later. Therefore, the test set consisted of four years of data: the 2009–2010 dengue transmission season through the 2012–2013 season.

#### Environmental data

NOAA environmental data were also posted on the Challenge website [20]. These data included surface measurements of temperature (i.e., daily maximum, minimum, average, and diurnal range) and precipitation, satellite remote sensing precipitation data, National Center for Environmental Prediction reanalysis data (i.e., reanalysis and assimilation of historical land surface, ship, rawinsonde, pibal, aircraft, satellite, and other data), and vegetation index data from the same time periods as the dengue case data. These climate data were provided in daily time series. The Challenge participants were given a choice as to whether or not to use these environmental data.

Our team downloaded the data, which were then aggregated by week to match the dates of the dengue case time series for each location. The specific climate variables tested for relevance to dengue dynamics were minimum and maximum temperature, satellite measurements of rainfall, and locally reported rainfall. Because the rainfall data were daily while the dengue data were weekly, the rainfall data were aggregated by week both for the satellite rainfall data and for the locally reported rainfall data. We found significant differences in rainfall data measured by these two methods. For reasons to be described in the Methods Section, it was found that the locally reported rainfall contributed the most to the dynamic variation in dengue cases.

### Dengue data pre-processing

As described above, data on total dengue cases were used for training and as prediction targets for both locations. The method of computing the total dengue cases was set by the Challenge organizers.

#### Special processing for Method of Analogues

The Method of Analogues is data-driven and the use of significant stretches of zero-dengue data would yield unreliable predictions. Therefore, the Iquitos dengue data were adjusted for some irregularities in reporting in the December 24-Jan 1 time frame for the training data. In these data sets, cases for the three-week period from the last part of December to the beginning of January were zero, although they were in what appeared to be the middle of the dengue transmission season. This effect was attributed to the possible closing of clinics around the Christmas holidays in these years. In this paper, we refer to this as the Christmas effect. To adjust for this effect, the total cases reported were kept constant, and cases from the post-holiday week were distributed over holiday weeks. The Iquitos test data set from 2009–2012 did not include zero values for the weeks December 24-Jan 1 so there was no Christmas effect and thus these data were not adjusted. The data from San Juan did not exhibit the Christmas effect.

#### Wavelets

When developing prediction models, it can be useful to smooth the data to minimize noise in order to allow important patterns to be modeled. A wavelet approach was used for smoothing the dengue data for use in Holt-Winters models. Wavelets are now commonly used in a variety of de-noising and compression applications, including JPEG images. Wavelets [21] employ a complete orthonormal basis (e.g., Daubechies, Haar, Symmlet) to represent functions, but then shrink and select the coefficients to achieve a sparse representation. This sparse representation allows for signal compression. The reconstructed signal is smoothed with respect to the original. Wavelet smoothing fits the coefficients for the given basis by least squares, and then discards the smaller coefficients. The sparser the representation (i.e., the smaller the wavelet order), the more smoothed the reconstructed signal is. The Haar wavelet is the simplest and its basis resembles a step function. The Daubechies wavelets extend the concept of Haar wavelets to functions with larger numbers of vanishing moments (i.e., orders). Symlets (also known as Symmlets) are mostly symmetrical forms of the Daubechies wavelets.

**Fig 1:**
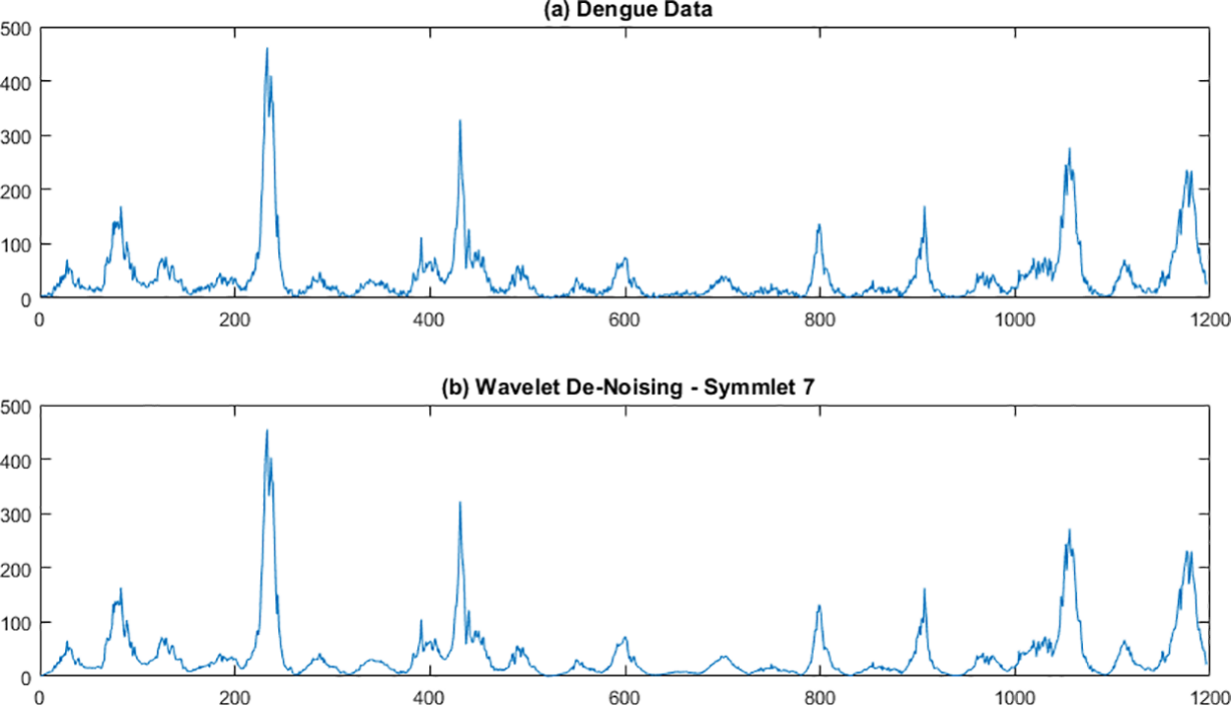
Symmlet 7 smoothing of San Juan dengue data. The abscissa is week number counting from the beginning of the data set.

**Fig 2:**
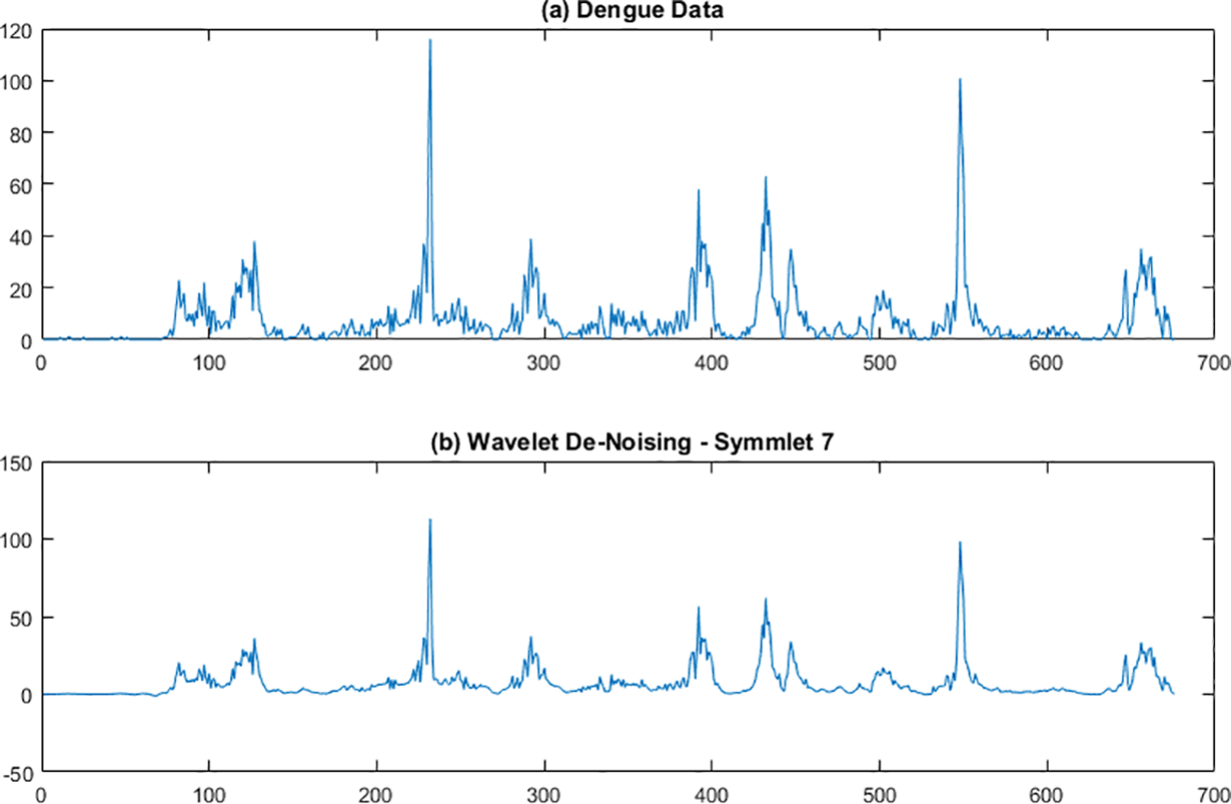
Symmlet 7 smoothing of Iquitos dengue data. The abscissa is week number counting from the beginning of the data set.

WaveLab850 [22] from Stanford University was used to apply the wavelet approach for smoothing the dengue data. Haar, Daubechies, and Symmlet basis wavelets of order 5–9 were used for smoothing San Juan and Iquitos data. Recall from above that smaller order numbers result in more smoothing of the data. As illustrated in Figs 1 and 2, Symmlet-basis order 7 wavelets provided the best balance between de-noising and keeping enough of the original shape when reconstructing the signal for San Juan (Fig 1) and Iquitos (Fig 2) data. Note that there were about 1200 weeks of data for San Juan, but about 700 weeks of data for Iquitos.

The wavelet smoothed dengue signals were subsequently used as input to certain Holt-Winters models, which are described in detail later in this paper. The Method of Analogues did not use the wavelet-smoothed data because smoothing the entire data stream can remove some nonlinear features that the Method of Analogues is able to exploit.

### Ensemble approach

#### Overview of the ensemble approach

In general, the wider variety of models used in the ensemble, the more likely each model may compensate for deficiencies in another, resulting in improved overall accuracy. In the present study, each ensemble model was created from several varieties of three distinct types of individual models: Method of Analogues models, Holt-Winters seasonal models, and historical models. These different types of models tended to complement each other in terms of their performance. The performance of these individual models varied so that the same model was not always the best, even from forecast to forecast. However, this is exactly the type of situation in which an ensemble approach would be beneficial.

There are three types of ensemble models, each predicting a different outcome variable:

- PHM–Peak Height Model—predicts the peak height (maximum number of weekly cases)
- PWM–Peak Week Model—predicts the week number in which the peak height occurs
- TNCM–Total Number of Cases Model—predicts the total number of cases in a dengue season (the 12-month period commencing with week with the location-specific, historical lowest weekly number of cases)

Each ensemble model is made of the 300 best performing models, including different numbers of Holt-Winters, Method of Analogues, and Historical models. For the ensemble model, we selected the best performing individual models based on past performance criteria described later. Results for individual models varied widely and differed from forecast to forecast. In general, individual models performed poorly and this was at least in part due to the noise found in this real dataset. The individual model results are described in the Supplemental Section (S1 File). The results for the ensemble models used in the Challenge will be described in the Results section. Each of the three individual component model types is described in the subsections that follow.

#### Multi-dimensional Method of Analogues

The Method of Analogues uses previous sequences of observations to predict future observations. It was originally developed by Edward Lorenz [23] for meteorological prediction and more recently used for influenza prediction [24]. Here we adapt the Method of Analogues to use sequences of both dengue cases and climate variable(s) to predict future dengue weekly cases (incidence).

It was necessary to determine which of the climate variables were relevant to dengue dynamics in the context of the Method of Analogues predictions. For this determination, we assessed the contribution of each climate variable’s contribution to dengue dynamics using transfer entropy [25]. Transfer entropy measures the contribution of a variable’s dynamics to the dynamics of another variable. Transfer entropy and time-delayed transfer entropy (i.e., using the climate variables from previous weeks) were calculated for all climate variables’ contributions to dengue dynamics. All of the temperature variables and the days of nonzero rainfall per week had very small transfer entropy. For both Iquitos and San Juan, maximum transfer entropy was observed between dengue and locally reported weekly total rainfall from 2 weeks before the incidence week.

The Method of Analogues uses the sequence of available observations leading up to the desired prediction date as a model. Sequences of observations in historical data closest to the model sequence (i.e., the “analogue sequences”) are found. If a prediction is desired *h* weeks ahead of the model, then the observations *h* weeks subsequent to the two-dimensional analogue sequences (dengue, locally reported rainfall) are averaged to arrive at the prediction. The length of the sequence of observations used for the model is designated *L*, and the number of analogue sequences used is designated *V*. In our implementation, the variables *L* and *V* are determined by a parameter sweep (described later in this section) on a subset of the data. This subset is then excluded from prediction testing. The variable *h* (the prediction horizon) was prescribed by the Challenge.

**Fig 3:**
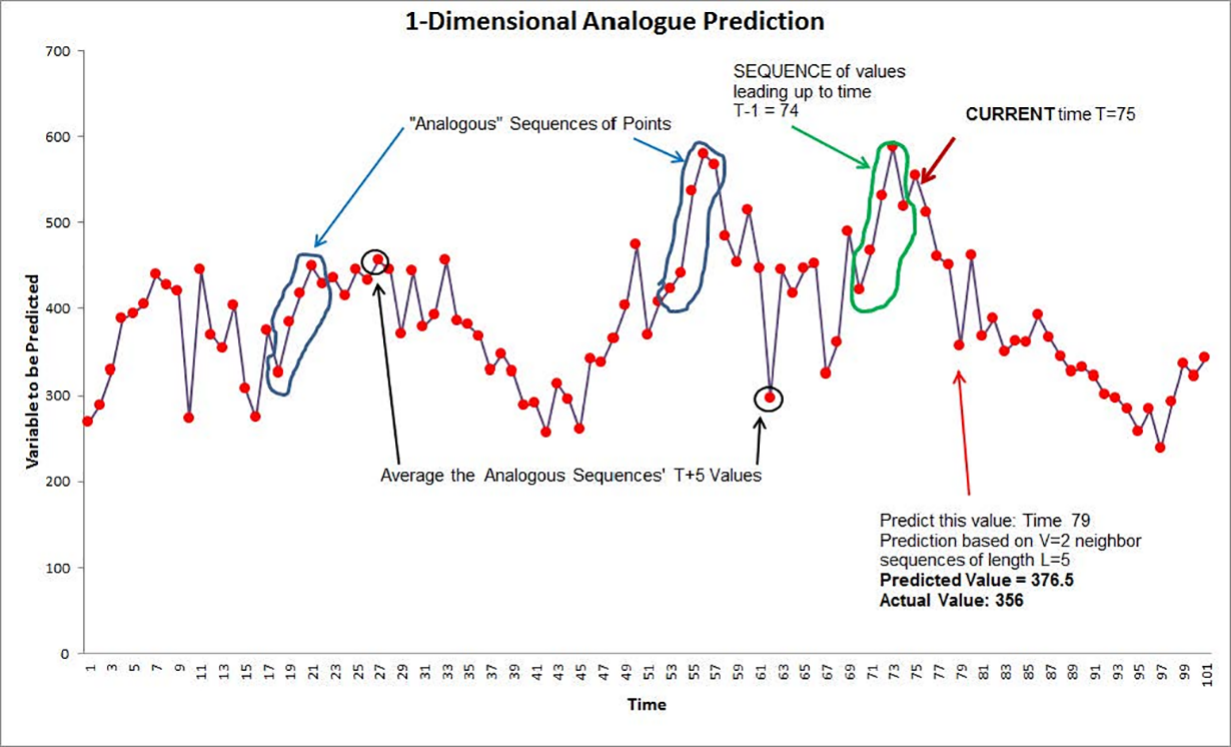
Example of a one-dimensional Method of Analogues prediction technique.

The underlying principle of the Method of Analogues is illustrated for the one-dimensional case in Fig 3. The red dots are values of fictitious disease incidence in a two-year period. We are making a *prospective* prediction of disease incidence at week 81 (green arrow); the prediction uses only the data available up to 5 weeks before the prediction. This prediction of the incidence value at week 81 (with parameters L = 5 and V = 2) assumes the current date is week 76 (and thus data are available only up to week 76). The black-circled sequence **S** of (L =) 5 observations prior to week 76 is used to find the (V =) 2 green-circled sequences in the historic data most analogous to S (i.e., closest in Euclidean distance to the sequence **S)**. The observations in the historic data 5 weeks ahead of the sequences (circled in black) are averaged to arrive at the prediction for week 81.

**Fig 4:**
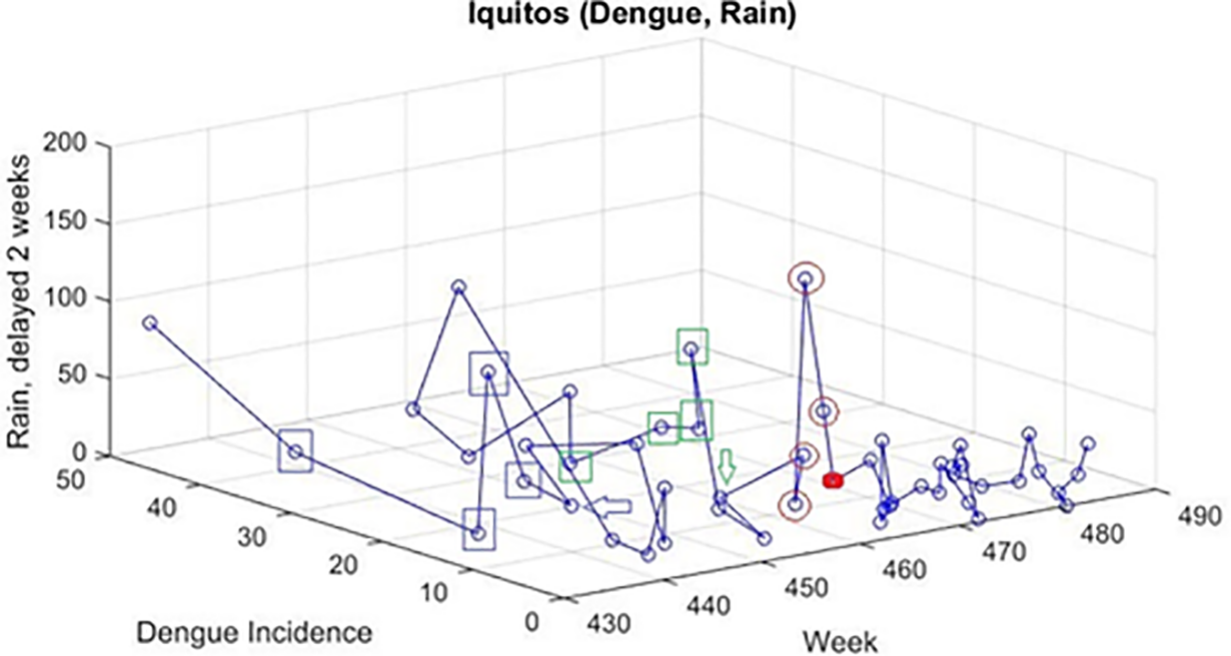
Example of using two-dimensional Method of Analogues to predict the value at the red dot at time t.

In the two-dimensional adaptation of the Method of Analogues, two-dimensional sequences were constructed using the dengue incidence as the first variable and the appropriately delayed weekly rainfall total as the second variable. Analogue sequences were found for the two-dimensional sequences and averaged to produce a predicted value. The dengue incidence only was taken from the analogous two-dimensional averaged sequence. Fig 4 shows an example of this two-dimensional approach in which the value at the red dot is predicted by finding two time-ordered sequences (V) of the four points (L) closest in Euclidean distance (in this context, $\sum_{(dengue,rain)in\;sequence}\sqrt{{\left({dengue}_{1}-{dengue}_{2}\right)}^{2}+{\left({rain}_{1}-{rain}_{2}\right)}^{2}}$) to these points, and then averaging the next point in these sequences. Both dengue incidence and rainfall are normalized (both scaled 0 to 1) to avoid numeric issues. In this figure, the two points designated by the arrows are used to predict the red dot; the two sequences (green and purple squares) are found to be closest to the red-circled sequence of points.

In order to find the parameters L (length of the prediction sequence) and V (number of sequences averaged for prediction), a parameter sweep was done for prediction of 4-week ahead incidence using only those incidence and rainfall values from the first 234 weeks (ending 12/23/2004) of the approximately 700 weeks of data for Iquitos and from the first 779 weeks (ending 4/16/2005) of the approximately 1200 weeks of data for San Juan. This parameter sweep means finding the values of L and V that have the smallest error (i.e., the squared sums of differences of the prospective predictions from the true values were smallest). For both locations, more than one pair of parameters (L and V) yielded the best prediction. For Iquitos, using L = 4 and V = 3 or L = 3 and V = 4 yielded similar results. For San Juan, using L = 3 and V = 8 or L = 8 and V = 3 provided similar results. Both sets of parameters were used in the predictions that served as inputs to the ensemble model. For the prediction made at week N, week N+1 through week 52 were predicted using only the data up to and including week N.

Transfer entropy was used to determine the climate variable that contributes most to dengue dynamics in these data. The transfer entropy (TE) is defined as follows:

$${TE}_{Y\to X}=\sum_{x_{i+1},x_{i},y_{i}}p(x_{i+1},x_{i},y_{i})\;\log_{2}\;\left\{\frac{p(x_{i+1}|x_{i},y_{i})}{p(x_{i+1}|x_{i})}\right\}$$

In this equation, X is the dengue sequence, Y is a climate sequence (e.g., either temperature or rainfall) that yields information about the dynamics of X, and ***p***(***x***_***i*+1**_,***x***_***i***_,***y***_***i***_) is the probability that the sequence of observations exhibited particular values of dengue at time *i*+1, dengue at time *i*, and the climate value at time *i*. Similarly, ***p***(***x***_***i*+1**_|***x***_***i***_,***y***_***i***_) is the probability that a particular value of dengue at time *i*+1 occurred, given the pair of observations of (dengue, climate) at time *i*. Lastly, ***p***(***x***_***i*+1**_|***x***_***i***_) is the probability of observing a particular value of dengue incidence at time *i*+1, given a particular incidence of dengue at time *i*. Transfer Entropy is a Kullback entropy [25] that measures the contribution of one sequence of observations to the dynamics of another. Unlike mutual information, Transfer Entropy can measure asymmetric relationships; for example sunlight contributes to the dynamics of the growth of a green plant but the growth of a green plant does not contribute to the dynamics of sunlight; there would likely be positive transfer entropy TE_sunlight->green plant_ but zero transfer entropy TE_green plant->sunlightt_. Because these relationships in Transfer Entropy are measured through the use of conditional probability densities computed directly from the sequences of observations, Transfer Entropy can capture complicated (e.g. nonlinear) effects of one variable on the other. In our example, if the effect of the sunlight is nonlinear, there will still be measureable Transfer Entropy from sunlight to green plant. Similarly, if the effect of a climate variable on dengue incidence is nonlinear, Transfer Entropy will still measure a positive effect. In this implementation of transfer entropy, the values of the probability densities were computed using fixed probability density rather than fixed radii because of the sparseness of the series.

For both locations, locally reported weekly total rainfall was the only variable to yield strongly positive transfer entropy. Therefore, the second dimension used for the Method of Analogues was locally reported rainfall. For Iquitos, using the data from 2000–2008, transfer entropy for the rainfall from two weeks prior to the dengue incidence data was higher than that for the rainfall from one week before. Thus, locally reported rainfall data with delays of two weeks were used as the climate variable in the two-dimensional Method of Analogues to obtain predictions in Iquitos. The transfer entropy from locally reported weekly total rainfall to dengue incidence was also highest using rainfall 2 weeks prior to the dengue incidence for San Juan.

The parameters for the number of sequences and the length of each sequence used for prediction were also recalculated from data through 2008 using a parameter sweep. For Iquitos, it was found that the pair of parameters L = 4 and V = 5 yielded the best predictions on this subset of the data (i.e., smallest RMS error). Thus, parameters for Iquitos were very strongly suggested as L = 4, V = 5. Together with delays of one and two weeks for the rainfall, the two-dimensional parameters for Iquitos were Model A: L = 4, V = 5, rainfall delay = 1 week; and Model B: L = 4, V = 5, rainfall delay = 2 weeks. To delay the rainfall by the prescribed number of weeks without omitting dengue incidence values from the sequence, the beginning of the entire rainfall sequence was padded with zeroes for the prescribed number of weeks. Ideally, true locally reported rainfall values for 1 and 2 weeks prior to the start of the dengue incidence data would have been more accurate than substitution of zeroes. However, this would have violated the rules for not inserting data other than that provided in the Challenge.

Method of Analogue predictions were obtained, as prescribed by the Challenge, for 4 to 52 weeks in advance of the prediction date (the date for which a prediction is sought). In the plots below, the predictions 52 weeks in advance are called “week 0” (i.e., they are made during week 0) predictions; those 4 weeks in advance are called “week 48” predictions. See the Supplemental Information (S1 File) for more results from the Method of Analogues. Examples of predictions for Iquitos and San Juan are shown in Figs A and B, respectively, in the S1 File.

#### Holt-Winters seasonal exponential smoothing method

In the late 1950s, basic exponential smoothing was proposed as one of the first time-series forecasting methods [26]. In its simplest form,

$$y_{t}=\alpha x_{t}+(1-\alpha )y_{t-1},\;0<t,\;0\leq \alpha \leq 1$$

where {*x*_*t*_} is the raw data sequence to be smoothed, {*y*_*t*_} is the output of the smoothing algorithm (also called the estimate), and α is the smoothing parameter. This equation is used to calculate *y*_*t*_ recursively (*y*_*t*−1_ is the most recent forecast) beginning with *y*_0_ = *x*_0_. An exponential decrease of weights is achieved when the value of α is set between 0 and 1, as can be seen in the following equation, which is based upon the recurrent equation above:

$$y_{t}=\alpha [x_{t}+(1-\alpha )x_{t-1}+{\left(1-\alpha \right)}^{2}x_{t-2}+{\left(1-\alpha \right)}^{3}x_{t-3}+\cdots +{\left(1-\alpha \right)}^{t-1}x_{1}]+{\left(1-\alpha \right)}^{t}x_{0}$$

In order to determine the operational value of α, an optimization stage is applied using the provided raw data series and minimizing the error between the raw data and estimated values. Hence, the exponential smoothing model can be fit to the input raw data [27]. In this work, two error functions are used to measure the deviation of the model predictions from the actual data:

$$\begin{array}{l} Root\;Mean\;Squared\;Error:\;RMSE=\sqrt{\frac{1}{n}\sum_{i=1}^{n}{\left(y_{t}-x_{i}\right)}^{2}}\\ Mean\;Absolute\;Relative\;Error:\;MARE=\sum_{i=1}^{n}\left|\frac{y_{t}-x_{i}}{y_{t}+1}\right| \end{array}$$

When the data exhibit enough seasonality resulting in an applicable periodicity, then Holt-Winters seasonal smoothing method may be used [27]. The following set of equations define the additive Holt-Winters model,

$$\begin{array}{l} y_{t+1}=a_{t}+b_{t}+s_{t-m}\\ a_{t}=\alpha (y_{t}-s_{t-m})+(1-\alpha )(a_{t-1}+b_{t-1})\\ b_{t}=\beta (a_{t}-a_{t-1})+(1-\beta )b_{t-1}\\ s_{t}=\gamma (y_{t}-a_{t-1}-b_{t-1})+(1-\gamma )s_{t-m} \end{array}$$

In the above equations, *α* is the smoothing factor, (0 < α < 1), β is the trend smoothing factor (0 < β < 1), γ is the seasonal change smoothing factor (0 < γ < 1). Similar to the basic exponential model approach, solving an optimization problem to find {α, β, γ} fits the Holt-Winters seasonal additive exponential smoothing model to the raw data. The optimization is performed in such a way that a given metric (RMSE or MAE) is optimized for the prediction one step ahead (t+1).

In our MATLAB implementation of the model, the initial conditions for {a_t_}, {b_t_}, {s_t_} are set to small numbers (0.1, 0.05, 0.05 respectively). The MATLAB optimization function **fmincon** is utilized for optimizing the {α, β, γ} parameters. For both Iquitos and San Juan, the additive Holt-Winters models were used because the seasonal variations were roughly constant throughout the seasons.

Different Holt-Winters models were developed using different periods of seasonality (51, 52, 53, 103, 104 and 105 weeks), and different ending weeks (1 through 52). Recall that ensemble models tend to perform better when they include a wider variety of individual models. Therefore, the input to half of these Holt-Winters models was the original data, while the input to the other half was the wavelet-smoothed data. Half of the models used RMSE and the other half MARE for parameter optimization. This yielded 1248 (= 6 * 52 * 2 * 2) different Holt-Winters models. The models were very sensitive to the different periods of seasonality and to the ending point of the training. Examples of how widely Holt-Winters component model results vary are shown in Figs C-J in the Supplemental Section (S1 File).

#### Historical models

For each of the two locations, we developed three simple historical models: 1) one for peak height, 2) one for peak week, and 3) one for total number of cases, in a dengue transmission season. The historical peak location model computes a histogram of peak location from the past data, and then predicts the peak location for which the histogram count is the highest. In cases where there are several bins with the same counts, one of the bins is chosen randomly because they are equally likely. If n bins are equally probable, then choosing one of them with a probability of 1/n would avoid bias. For peak height and total number of cases, the same approach is used; however, because the bins are wider than 1, the predicted value is the average of the bin’s lower and upper limit. For example, if the histograms’ highest count for peak height is 2, and if bin 2 corresponds to 100–200, the predicted value will be (100+200)/2 = 150.

The histograms were computed from the training data (for Iquitos, 2000–2009; for San Juan, 1990–2009) for each of the three variables. The histograms for San Juan are shown in Figs K-M, and the histograms for Iquitos in Figs N-P in the Supplemental Information S1 File. When there is one maximum in the histogram (see Figs K, M, N in the Supplemental Information S1 File), the middle of this bin will be always predicted by the historical model (e.g., 28 for peak week for San Juan). In case there is more than one maximum (see Figs L, O, P in the Supplemental Information S1 File), each of the middle bin values is predicted with a probability 1/count (i.e., 0.5 probability for peak Value for San Juan). The historical model for the total number of cases for Iquitos is an especially poor model, as there are 8 bins with the same height (height of 1). This means that this model will predict one of those bins with a probability of 0.125. This illustrates the great difficulty of making predictions for Iquitos. Overall, historical models did not perform well but they were a valuable complement to the other two types of models. Historical models tended to perform best at the beginning of the season when very little is known about the dengue cases for that season and predictions are being made up to 48 weeks in advance.

#### Choosing individual classifier weights for the ensemble

Recall that there were three forecast targets defined by the Challenge for each of the two cities: 1) peak height, 2) peak week; and 3) the total number of cases, in a dengue transmission season. Forecasts of these targets were made for San Juan, Puerto Rico, and Iquitos, Peru. Forecasts were required for all three of the forecast targets for both locations every four weeks (predictions were made in weeks 0, 4, 8, 12, 16, 20, 24, 28, 32, 36, 40, 44, and 48). Each forecast consisted of a point estimate of the forecast target and a binned probability distribution where probabilities were required by the Challenge to be assigned to predefined bins specific to the target type. These forecasts were made over a four-year period. For each of the target—location pairs, an ensemble model was developed for each of the four years. In order to generate an ensemble forecast, the predictions of the individual models chosen for the ensemble were combined by identifying the top performing models and then weighting the strength of their contribution to the ensemble forecast by the level of their performance on the training data. It is important to note that there was considerable variation in which individual model performed best for a given target-location pair, even from one forecast to the next. Thus, the best performing individual models varied so there was no consistently best component model. The ensemble approach thus adds stability to model performance, which is one reason why the ensemble approach is useful [14].

For a given forecast, the 300 best performing component models (to be used in the ensemble model) were chosen from among the set of Holt-Winters models whose training data ended anytime between week 1 of the previous year and the week prior to the forecast time, the set of predictions made by the Method of Analogues whose training data ended prior to the forecast week, and at most one historical model. The number of Holt-Winters models considered for use in the ensembles for both locations varied from 1248 for the week 0 forecasts to 2400 for the week 48 forecasts. The number of the Method of Analogues predictions considered for the Iquitos ensembles varied from 2 for the week 0 forecasts to 26 for the week 48 forecasts. The number of the Method of Analogues predictions considered for the San Juan ensembles varied from 4 for the week 0 forecasts to 52 for the week 48 forecasts. Historical models tended to perform best at the beginning of the forecast year but with a subsequent decrease in performance so that they often were not included in later forecasts.

To identify the top performing models, a metric was developed to capture the expected performance of the individual model forecasts. As discussed above, each of the individual models is uniquely identified by a set of parameters. It is reasonable to expect that the performance of a given model would be highly correlated with the performance of another model trained in the same way with the same set of parameters and tested on a prior year of data. Therefore, the chosen metric was the predictor of the expected error of the given model, which we derived as the average error of forecasts made at the same month over the four previous years by models that were trained identically. As an example, for a fixed forecast target and location, we may compute this metric for the forecast *F*_*A*_ made by model *A* defined by the set of parameters *P* at month *M* of year *Y*, by first training models *A*_*1*_, *A*_*2*_, *A*_*3*_, and *A*_*4*_ using the same set of parameters *P* to make forecasts for the years *Y*-1, *Y*-2, *Y*-3, and *Y*-4 respectively. Then, we obtain forecasts *F*_*1*_, *F*_*2*_, *F*_*3*_, and *F*_*4*_ made by these four models at month *M* of years *Y*-1, *Y*-2, *Y*-3, and *Y*-4 respectively and compute the respective errors *E*_*1*_, *E*_*2*_, *E*_*3*_, and *E*_*4*_. Finally, we take the average of *E*_*1*_, *E*_*2*_, *E*_*3*_, and *E*_*4*_ and assign it as the expected error of the forecast *F*_*A*_ from model *A*. The 300 models (regardless of type) with the lowest expected error for their forecast for a given month were selected to contribute to the ensemble forecast for that month. We chose 300 because our analysis showed diminishing returns in general when more models than this were included in the forecast.

To enable the stronger models to have a larger contribution to the ensemble forecast, weights were computed based on the relative performance of the models. The worst performing model of the 300 selected was assigned a weight of one. The other models were then assigned a weight determined by the inverse of the ratio of their expected error metric to the expected error metric of the worst model. The weight assigned to a specific model determined the number of times that it “cast a vote” using its forecasted value. The median value of all the votes cast was used as point estimate for the ensemble forecast for the month. The normalized distribution of the votes that were cast by the top models was used as the binned probability distribution of the forecast.

Note that there was a different ensemble model for each location-forecast pair and for each season. The 300 top performing component models used in the ensembles varied considerably. In general, the Holt-Winters types of models dominated. Recall that each Holt-Winters model used different parameters, including periods of seasonality and training end points, and these parameters used different optimizations. Holt-Winters models were very sensitive to different periods of seasonality and to training end points. The Method of Analogues predictions did much better on both seasonal total dengue case forecasts and the peak height forecast for San Juan than they did for the other 4 location-forecast pairs. This is reflected in the proportion of their predictions that were selected for inclusion in the top 300. For these three, a high proportion were selected for most forecasts especially early in the year and in some cases all available models were selected. However, for the other 4 location-forecast pairs, a very low number were selected if any. As seen in the Supplemental Information (S1 File), the individual component models often performed poorly. The ensemble approach is designed to perform better by combining the diverse individual models in a way that takes advantage of the differences in their past performances for different situations [14].

## Results

As determined by the Challenge organizers, each forecast consisted of a point estimate and a binned probability distribution where probabilities were required to be assigned to predefined bins for each target type. The sum of the probabilities across the bins had to equal one. Forecasts were made every four weeks starting at week 0 (i.e., before the year started) resulting in a total of 13 forecasts per year. Recall that the test set consisted of four years of data: the 2009–2010 dengue transmission season through the 2012–2013 season. The data in this test set were not used at all during the development of the ensemble methods, but all data up to and including the week the forecast were made were available for use as inputs to the models when making the forecasts.

Point estimates were evaluated using the mean absolute error (MAE), which is the mean absolute difference between predictions and observations over a set of data points.

$$MAE=\frac{1}{n}\;\sum_{i=1}^{n}\left|{\hat{y}}_{i}-y_{i}\right|$$

The binned probability distributions *P* were evaluated using the logarithmic scoring rule as a means of comparing the confidence of the forecasts. This score is defined to be the logarithm of the probability assigned to the bin that contains the observed outcome, *i*.

$$S(P,i)=ln(P_{i})$$

An example of the binned probability distributions is given in Table 1.

**Table 1:** Example of the probability distribution portion of the forecasts.

	2009/2010_wk0	2009/2010_wk4	2009/2010_wk8	…
p(0< = season_incidence<100)	0.034722	0.02	0.014877	
p(100< = season_incidence<200)	0.069333	0.045	0.034384	
p(200< = season_incidence<300)	0.163278	0.185	0.134356	
p(300< = season_incidence<400)	0.086639	0.1675	0.161178	
p(400< = season_incidence<500)	0.145972	0.17	0.244082	
p(500< = season_incidence<600)	0.126194	0.0975	0.170932	
p(600< = season_incidence<700)	0.089111	0.065	0.058767	
p(700< = season_incidence<800)	0.084167	0.065	0.056329	
p(800< = season_incidence<900)	0.089111	0.08	0.05389	
p(900< = season_incidence<1000)	0.027306	0.025	0.019753	
p(1000< = season_incidence)	0.084167	0.08	0.051452	

**Fig 5:**
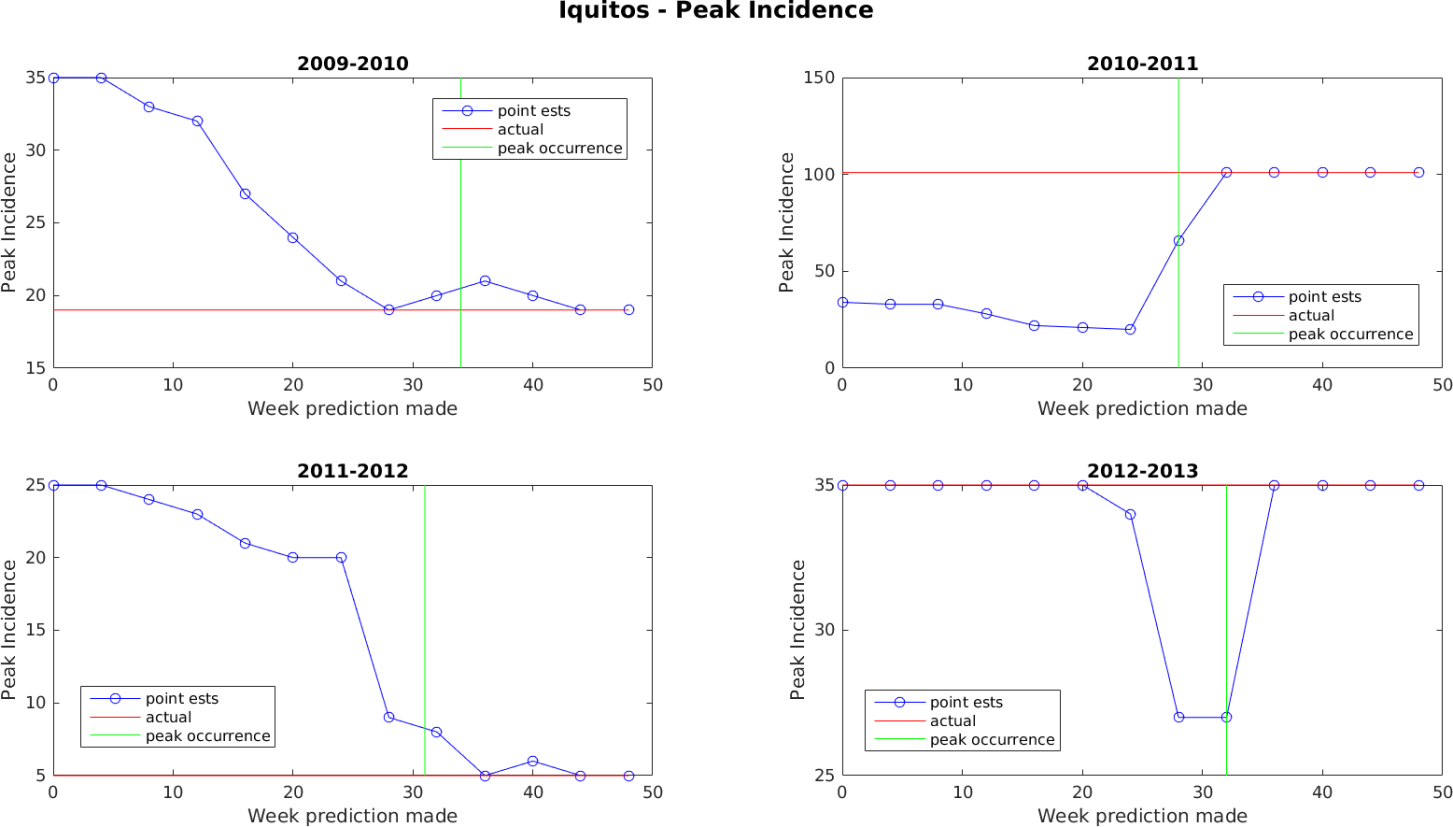
Point estimates of peak incidence for Iquitos.

**Fig 6:**
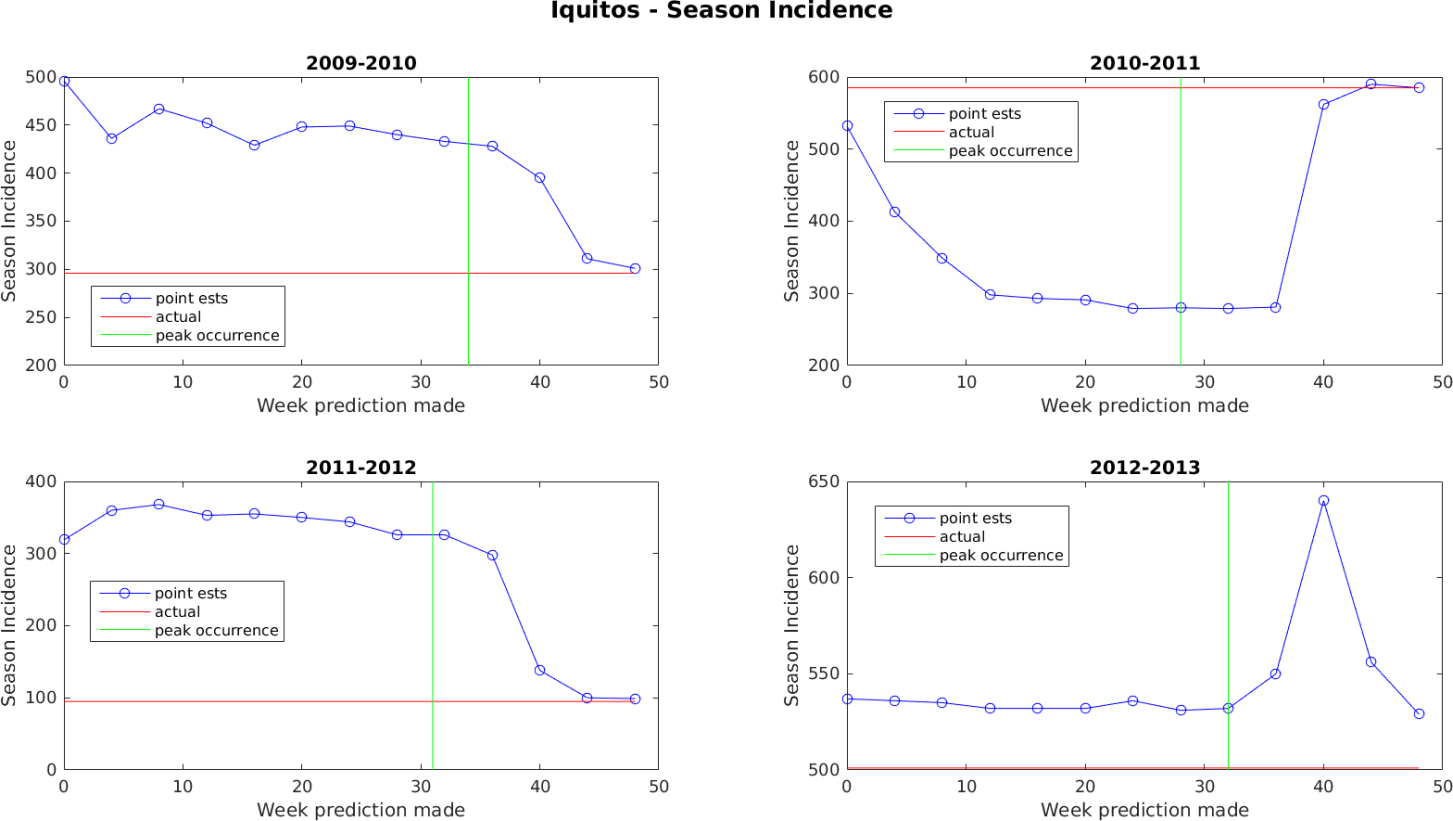
Point estimates of seasonal dengue incidence for Iquitos.

**Fig 7:**
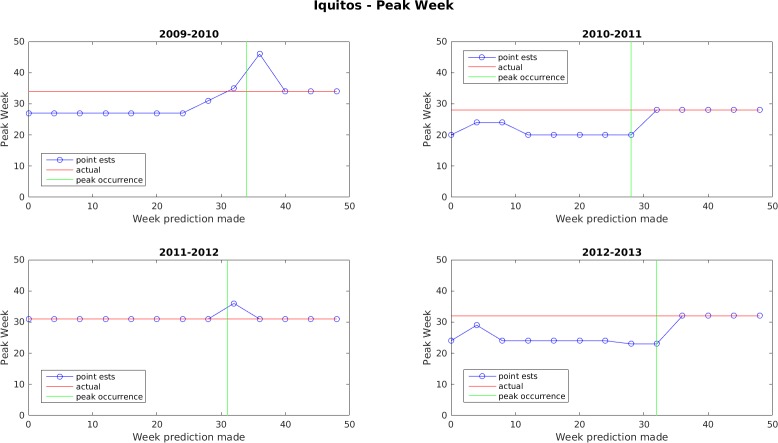
Point estimate of peak dengue week for Iquitos.

**Fig 8:**
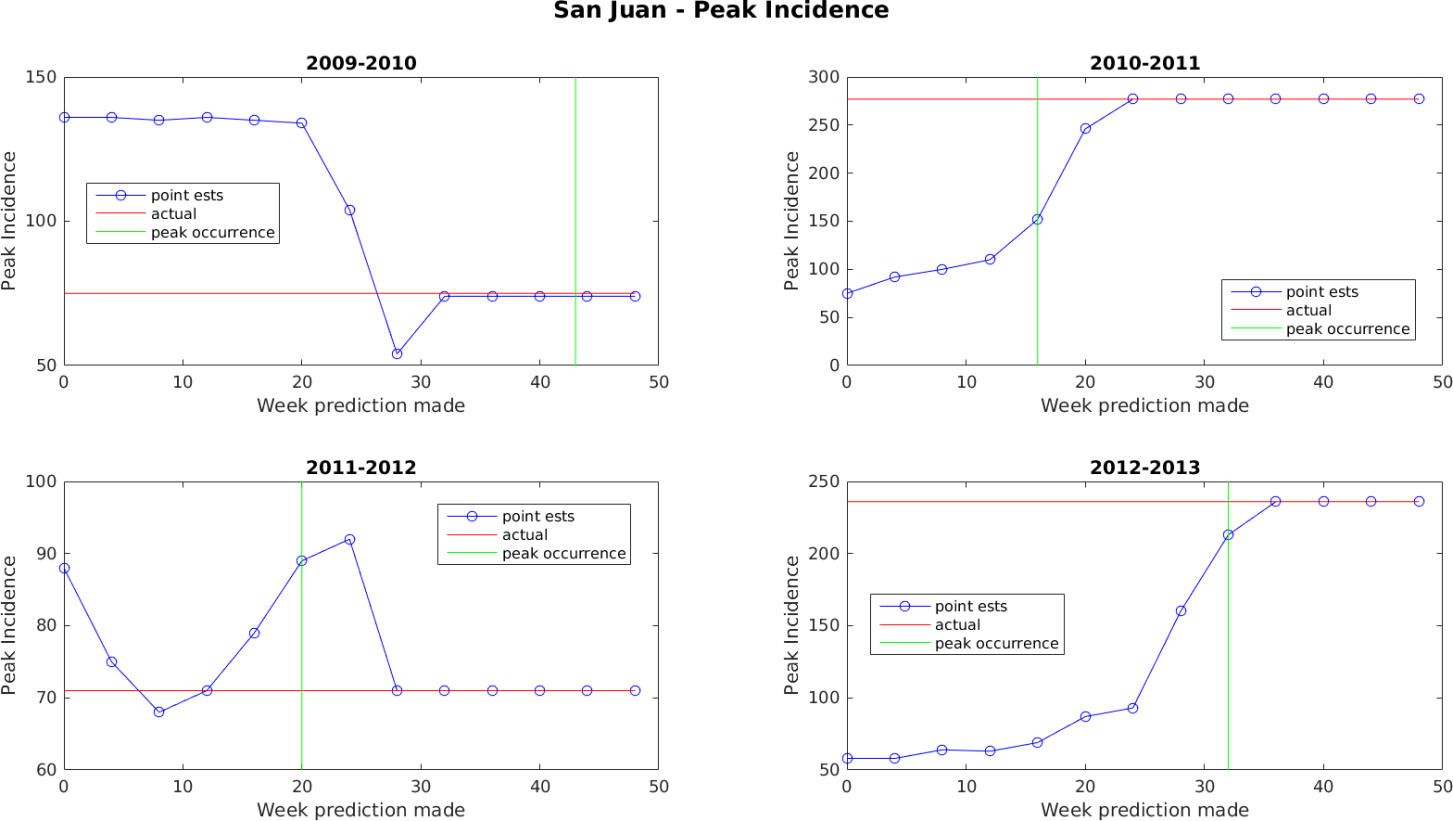
Point estimate of peak dengue incidence for San Juan.

**Fig 9:**
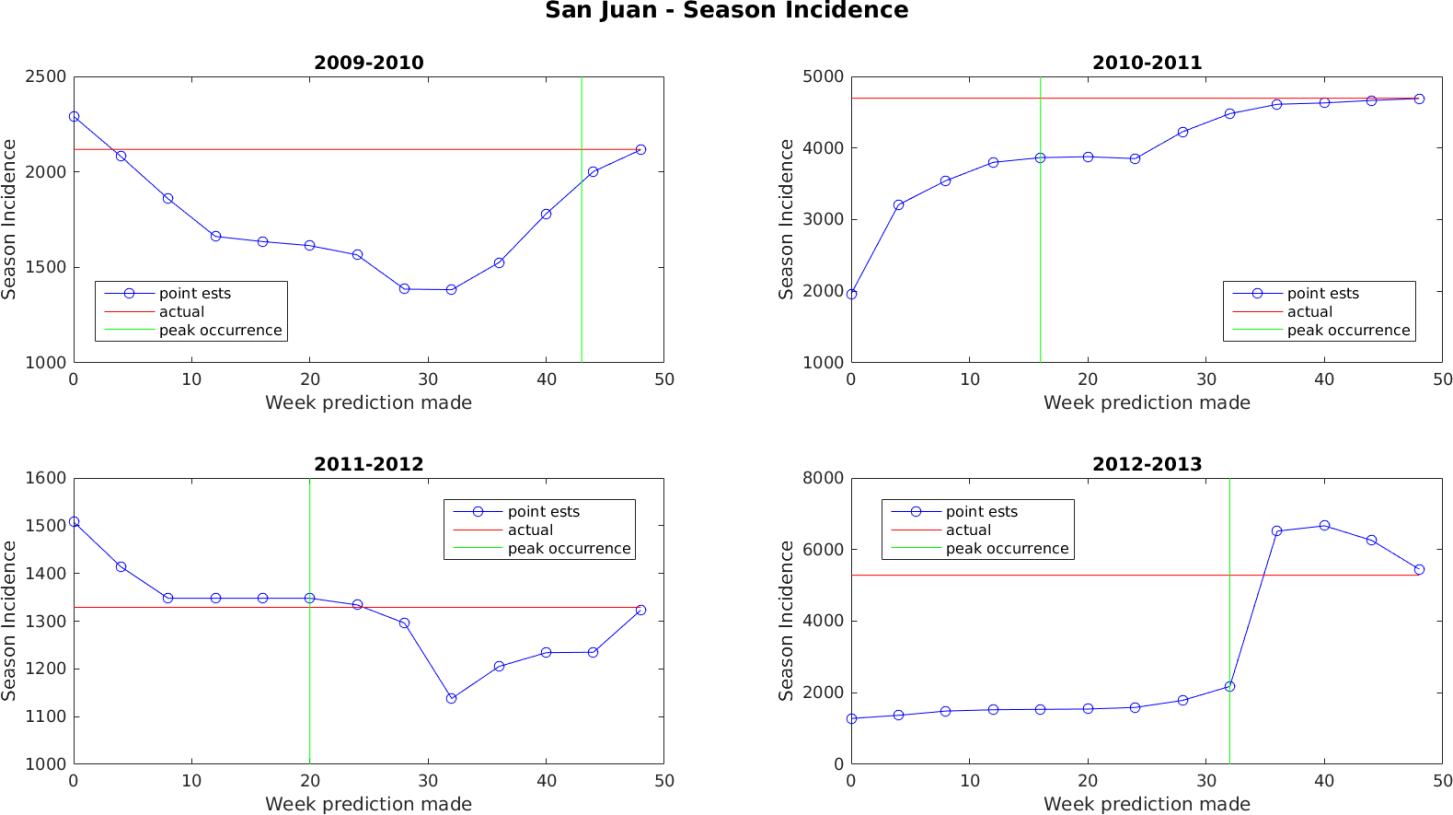
Point estimate of seasonal dengue incidence for San Juan.

**Fig 10:**
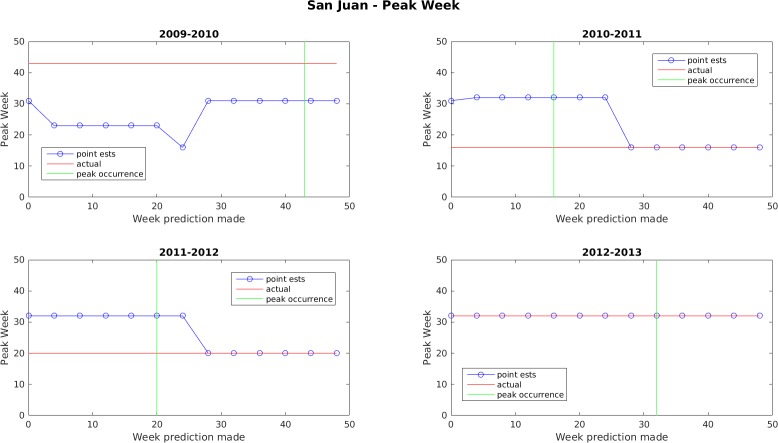
Point estimates of peak dengue week in San Juan.

**Fig 11:**
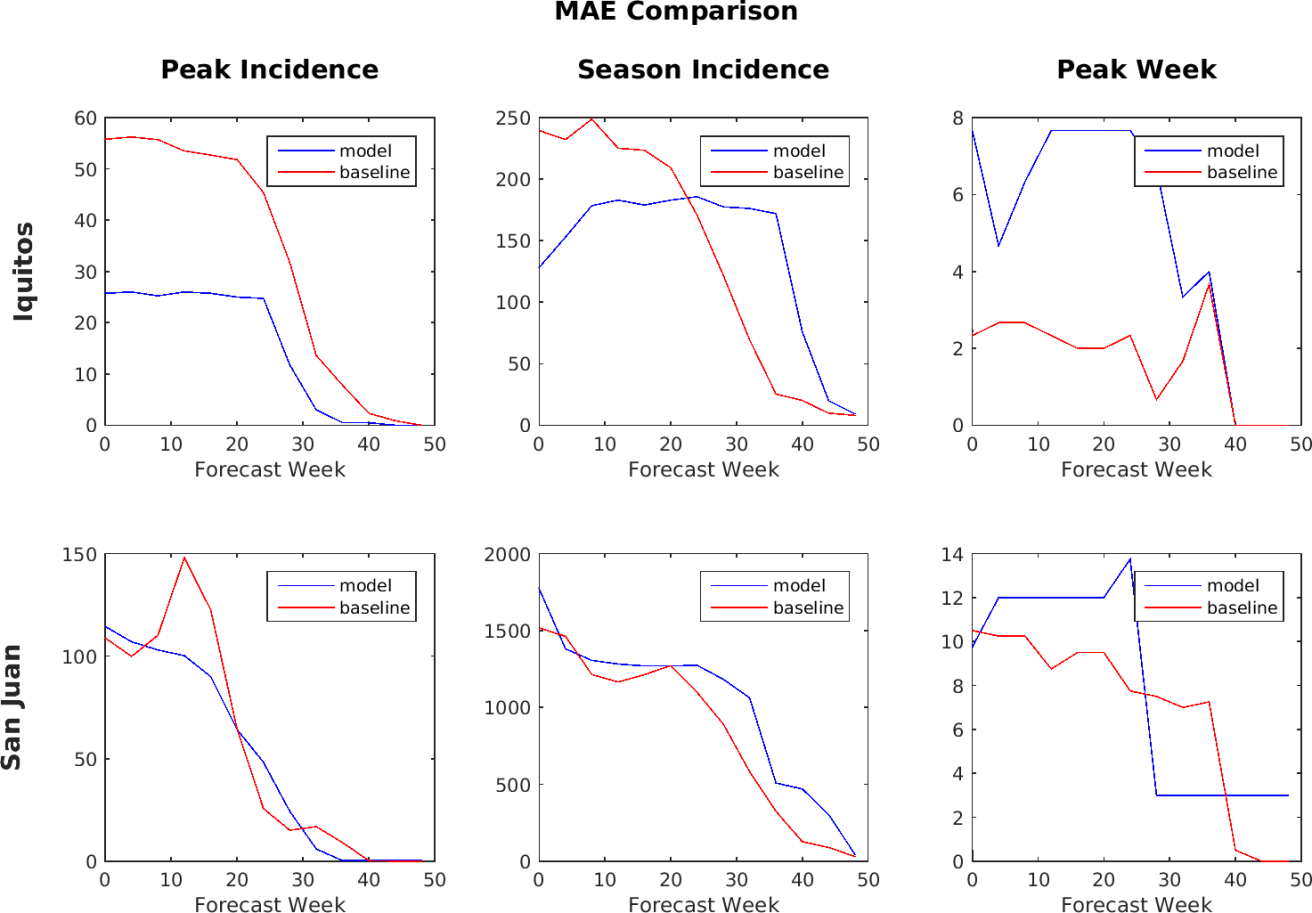
MAE comparison of Ensemble vs. SARIMA (lower number is better).

Figs 5–11 display the point estimates of the ensemble forecasts in relation to the actual values ("ground truth") over the 4 years of the test set for each forecast target and each location. The abscissa in these plots (i.e., forecast week) is the week number when the prediction was generated.

In general, the forecasts for peak week timing and maximum weekly incidence converge to the correct values before or shortly after the occurrence of the peak. The notable exception to this is the 2009–2010 dengue transmission season for San Juan. For this season, the true peak occurred very late in the year, but there was an earlier peak with a height that was only 1 case less than the true peak. The ensemble latched on to this first peak and did not accumulate enough evidence before the year ended to switch to the higher peak. The total season incidence forecasts do not converge as quickly because the true value is always being altered for a month beyond the last forecast made.

In order to compare the results of the submitted models to those of a baseline model, a seasonal autoregressive integrated moving average (SARIMA) model was developed by the Challenge organizers. This SARIMA model is denoted by *ARIMA*(1,0,0) × (4,1,0)_52_. This is the comparison by which the Challenge organizers scored the competing models. The non-seasonal component consists solely of an order 1 autoregressive (AR) model, and the seasonal component consists of an AR model of order 4 with a single difference term. The period of their model is 52 weeks.

Fig 11 shows a comparison of the MAE of the point estimates for the six forecast target/location pairs of the ensemble predictions and the baseline model predictions. These are predictions using the test set of data that was kept separate from the training data. The MAE is averaged over the four seasons in the test set. That is, each point on the graph represents an average of the predictions made for that week over 4 years (i.e., dengue seasons). For example, the week 0 performance is the average of the week 0 performance in each of these four years. When comparing MAE, a lower number (smaller value) is better. By this metric, the SARIMA model generally performed better for peak week prediction in Iquitos and for both peak week and season incidence predictions in San Juan, while the ensemble model generally performed better for peak incidence and season incidence predictions in Iquitos and peak incidence predictions in San Juan. For Iquitos season incidence, the ensemble prediction performed better than the Challenge baseline model up until about week 20, after which the baseline model performed better.

**Fig 12:**
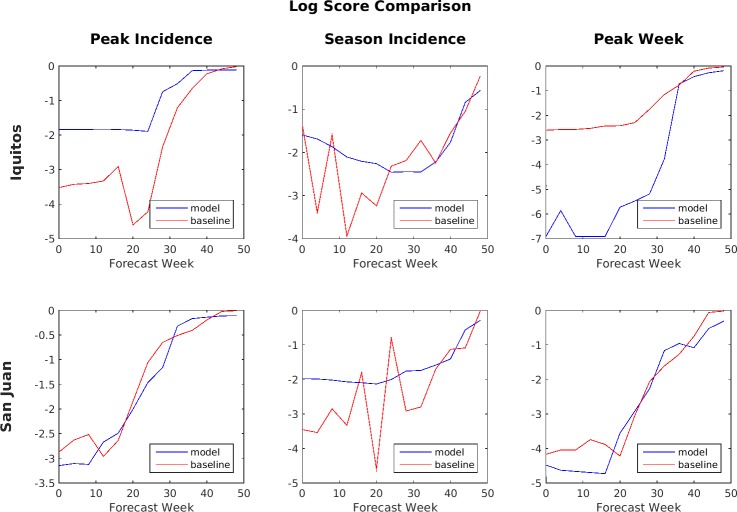
Log score comparison of ensemble vs. SARIMA (higher number is better).

Fig 12 shows a comparison between the ensemble model and the baseline SARIMA model of the averaged log scores of the binned probability distributions for the six target/location pairs. The average is taken over all four seasons in the test set. When comparing log scores, a higher (e.g., less negative) score is better. By this metric, the SARIMA model generally performed better for peak week timing predictions in both Iquitos and San Juan, while the ensemble model performed better for peak weekly incidence and total season incidence predictions in Iquitos and total season incidence predictions in San Juan.

Accuracy during the early part of the season is considered much more important than the accuracy later in the season because these are the predictions most likely to impact decision making. As a result, the Challenge organizers compared models primarily based on the performance of their forecasts made during weeks 0 through week 24. During this period, the SARIMA model performed better by the MAE metric for peak week timing prediction in Iquitos and for both peak weekly incidence and total season incidence predictions in San Juan, while the ensemble model performed better for peak weekly incidence and total season incidence predictions in Iquitos and for peak weekly incidence predictions in San Juan. For the log scores, the SARIMA model performed better for peak week timing predictions in Iquitos and for both peak week timing and peak weekly incidence predictions in San Juan, while the ensemble model performed better for peak weekly incidence and total season incidence predictions in Iquitos and for total season incidence predictions in San Juan.

The evaluation metric chosen by the Challenge organizers for this project was the average log score of the probability distributions of the forecast across weeks 0 through 24 for all 4 years. However, the 2011–2012 forecast season was excluded by the Challenge organizers from Iquitos peak week comparisons because there were three peaks instead of one. Our predictions of peak week were very good for Iquitos for the excluded 2011–2012 season. The fact they were not taken into consideration when computing the scores substantially contributed to our poor overall scores for the Iquitos peak week predictions. Table 2 provides these scores for the ensemble model with a comparison against the performance of the Challenge baseline SARIMA model.

**Table 2:** Comparison of average log scores across months and years.

Forecast Target	SARIMA Model	Ensemble Model	Improvement
Iquitos–Peak Height	-3.6	-1.8	50%
Iquitos–Season Incidence	-2.7	-2.0	26%
Iquitos–Peak Week	-2.5	-6.4	-156%
San Juan–Peak Height	-2.4	-2.6	-8%
San Juan–Season Incidence	-2.9	-2.0	31%
San Juan–Peak Week	-3.9	-4.2	-8%

By the average log score metric, the ensemble method performs the best overall when compared to SARIMA for predicting season incidence (26% and 31% improvement for Iquitos and San Juan, respectively). Its performance on peak incidence is mixed (50% improvement for Iquitos, but 8% decrease for San Juan). The performance of the ensemble method on peak week is disappointing (156% worse than SARIMA for Iquitos, and 8% worse than SARIMA for San Juan). The poor relative performance for Iquitos peak week timing predictions is due to a combination of two factors: the SARIMA model doing extraordinarily well (i.e., better than all models submitted to the Challenge) for this forecast target and a lack of diversity in the ensembles used for these predictions. These factors will be discussed in more detail below. Also note that the 2011–2012 forecast season was excluded from Iquitos peak week comparisons by the Challenge organizers when scoring the Challenge submissions because that season did not have a unique peak but instead had three peaks. However, our ensemble model successfully identified the first peak as the primary peak for that season. If that season had been included, the ensemble model score would increase from -6.4 to a more respectable -4.9.

For the Iquitos peak incidence and total season incidence forecast targets, the ensemble model outperformed not only the baseline SARIMA model but also all the models developed by other teams participating in the Challenge. Of the 16 competing models, it was the only model that had the top score for two forecast targets.

**Fig 13:**
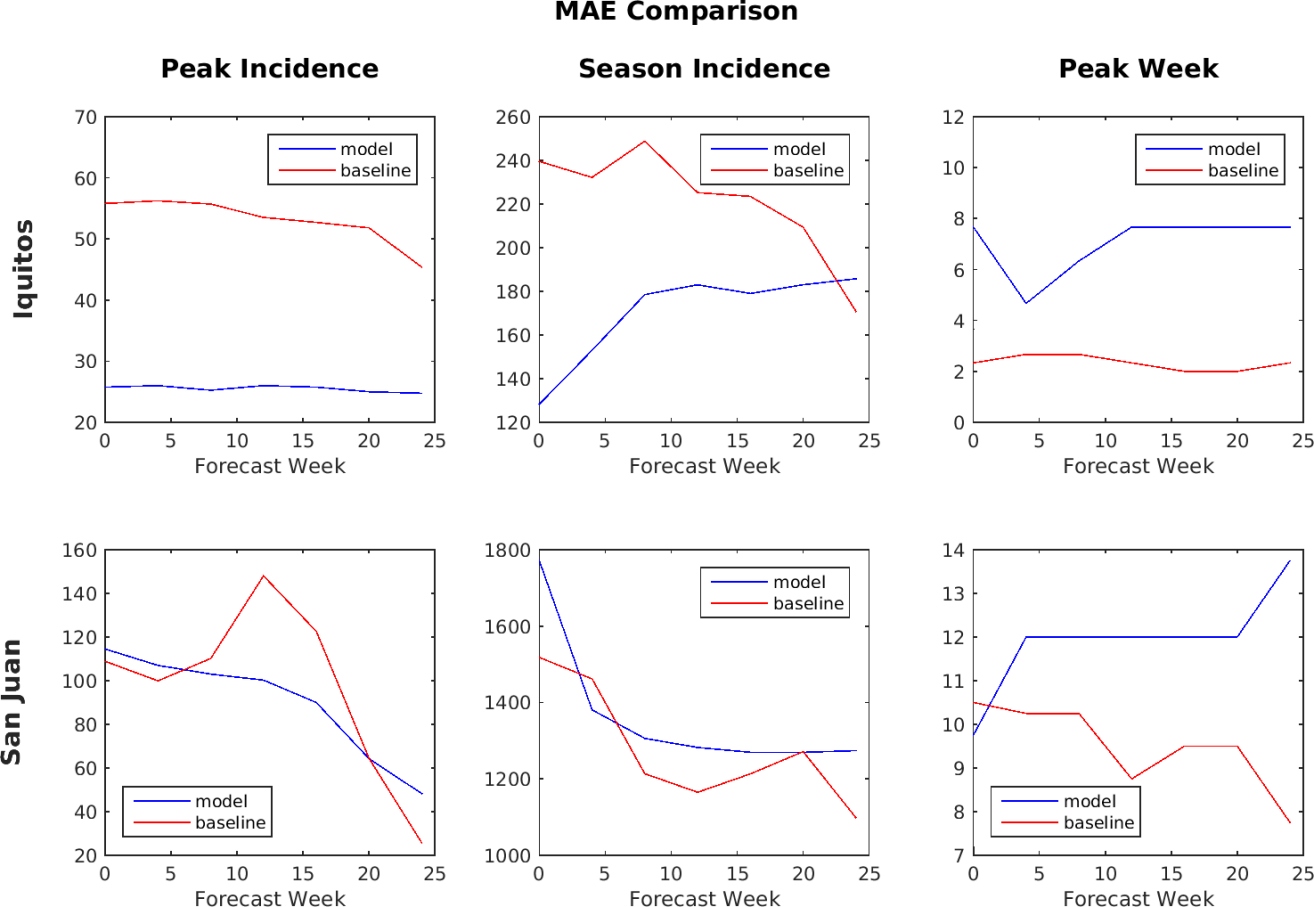
MAE comparison based on lead-time for Ensemble vs. SARIMA (lower numbers are better).

**Fig 14:**
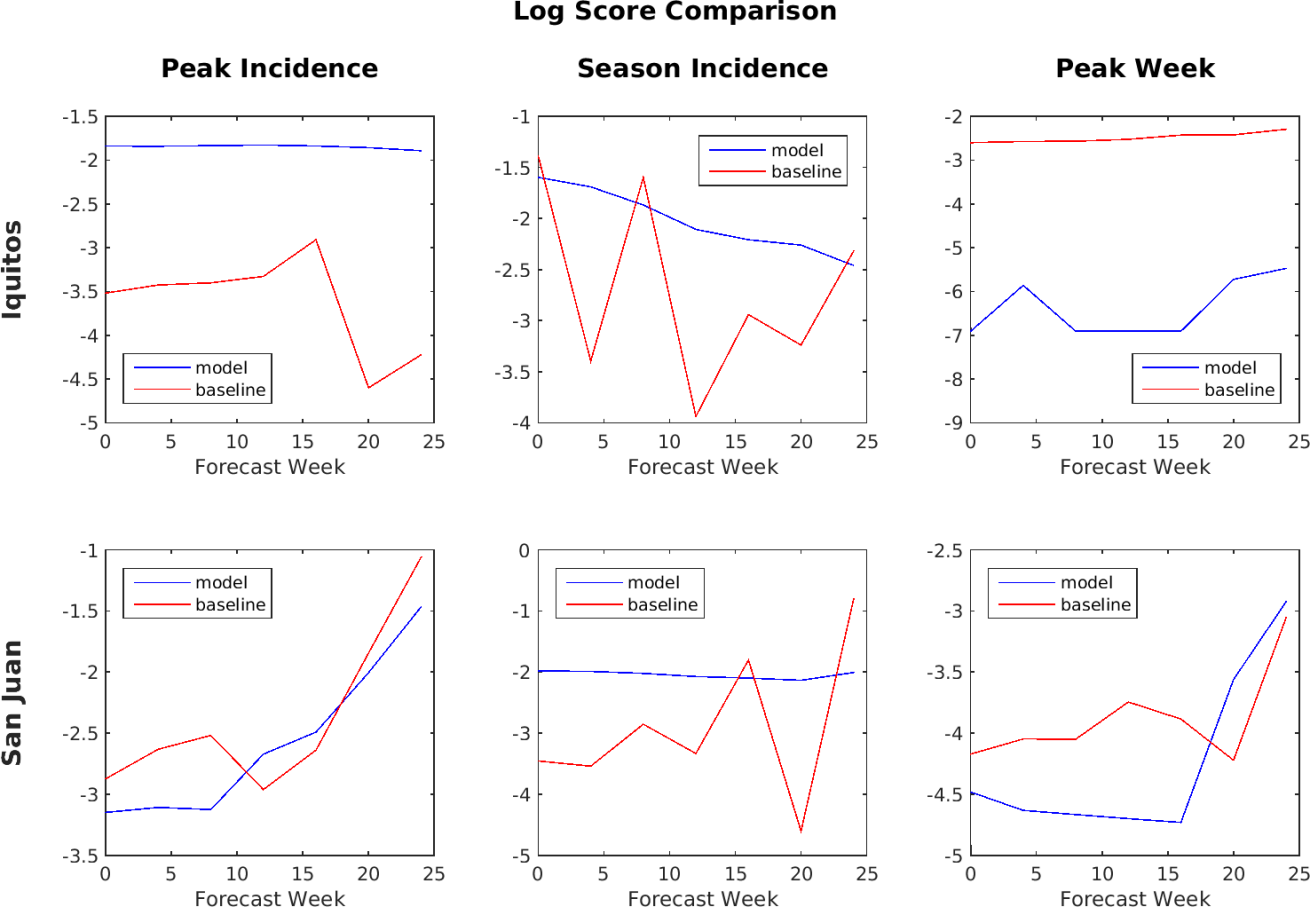
Log score comparison based on lead-time for ensemble vs. SARIMA (higher numbers are better).

Another way to compare performance between the models is by using lead-time because the timing of the peak incidence varied from as early as week 16 to as late as week 43 in the test data set. For this analysis, all forecasts made within 1 month before an actual peak were lumped together, those made between 1 and 2 months before were lumped together, and so forth. Also note that, for peaks that occurred earlier in the season, values for some of the longer lead times would be unavailable and so only the available values were averaged. Figs 13 and 14 show the comparison based on lead-time between the ensemble model and the SARIMA model.

In general, performance behavior is similar to the previous comparison, but it is interesting to note that the performance of the ensemble model in the majority of cases either stays constant or actually improves slightly as lead-time increases. This may just be a statistical anomaly as there are fewer data points as lead time increases, but there could be an underlying cause, which we may attempt to uncover in a future study.

## Discussion

As determined by the Challenge organizers, model accuracy was assessed for three different types of predictions for two different locations (Iquitos and San Juan): 1) peak height (i.e., maximum weekly number of cases during a transmission season; 2) peak week (i.e., week in which the maximum weekly number of cases occurred); and 3) total number of cases reported during a transmission season. The Challenge organizers considered each forecast of a point estimate and a binned probability distribution in which the probabilities were assigned to predefined bins for each of the three types of predictions listed above. As described earlier, prediction accuracy was scored for point estimates by using MAE and for probability distributions by using the logarithm of the probability assigned to the bin with the observed outcome. Note that the data chosen by the Challenge organizers was real surveillance data and therefore quite noisy (e.g., Figs 1 and 2).

Overall, our ensemble model performed the best for predicting two of the targets for Iquitos, Peru: the total number of dengue cases in a transmission season and the peak height (i.e., maximum weekly number of dengue cases). For these particular targets, our ensemble model significantly outperformed the baseline SARIMA model as well as all of the other models submitted to the Challenge. For San Juan, Puerto Rico, the accuracy of our predictions for the total number of seasonal dengue cases and the peak weekly number of dengue cases, predictions were mixed depending on which metric was used for the comparison. One possible factor in these performance results may involve the technique used by the Challenge organizers for estimating the additional San Juan cases. Recall that, for San Juan only, the Challenge organizers derived weekly case numbers by multiplying the number of untested cases by the rate of lab-positive cases among the tested cases. So, the San Juan dengue data are based on the assumption that the untested specimens would have had the same percentage of dengue positives as the tested specimens. It is possible that this assumption is incorrect. If so, this could have impacted the model performance results for San Juan.

As was mentioned above, the results for the peak week timing predictions were disappointing, especially for Iquitos. Part of the large disparity in the scores for Iquitos was due to the Challenge baseline SARIMA model doing extraordinarily well on this particular data set. That is, the baseline SARIMA model outperformed all of the models submitted to the Challenge for this forecast target, outperforming the top submitted model by approximately 20%. The other major factor was that our binned probability distributions were very sparse due to the large number of prescribed bins for the peak week timing forecast target and our method for forming the distributions from the weighted votes of the ensemble models. The peak week timing forecast target had 52 prescribed bins while the other two forecast targets only had 11 prescribed bins each. There was also much more agreement (i.e., the majority of the models making similar predictions) early in the season among the ensemble of models for Iquitos peak week timing predictions than for the other forecast targets. As mentioned above, we formed the required probability distribution from our ensemble of models by placing the models’ “votes” into the appropriate prescribed bins and normalizing. Ideally, this would result in a smooth distribution of values with a peak at the bin containing the ensemble point prediction. However, due to the reasons mentioned above, the peak week distributions were far from this ideal. To demonstrate this, we computed the average percentage of the probability bins that received votes during the first half of the season and these results are summarized in Table 3. Note that the percentage is significantly lower for the peak week predictions than the others and that this effect is much worse for Iquitos than for San Juan. These numbers show how sparse the distributions were for the peak week predictions and are highly correlated with the log scores. Ideally predictions that are off by only a few weeks should still score relatively well by the log score metric, but the sparse distributions prevented this from happening.

**Table 3:** Mean percentage of probability bins receiving votes in weeks 0–24 by forecast target.

Location	Season Incidence	Peak Height	Peak Week
Iquitos	87%	56%	**14%**
San Juan	70%	65%	26%

For prediction, different individual models may work well in certain situations but not in others. In our case, there was little consistency so that an individual type of model did not performed best in every prediction, even from one forecast to the next. Thus, an ensemble approach might be expected to provide better predictions that individual component models [see, e.g., 14].

While using ensemble models to predict disease outbreaks is not novel (e.g., [16], [19]), the ensemble model building technique described herein used a new approach of choosing the best performing individual component models and assigning them weights based on past performance for a given week number in the previous 4 years of data. The Method of Analogues model is novel in that it uses analogue sequences for two variables: weekly dengue incidence and weekly rainfall data. A variety of Holt-Winters models (including some with and some without wavelet smoothing) were used. While the Historical models are the simplest and least effective of these types of models, they still provided nearly the best performance early in the season (week 0).

Our ensemble approach could be improved in a number of ways. For example, the Holt-Winters part of the ensemble could possibly have been modified to give a better peak week prediction performance. The construction of a multi-dimensional Method of Analogues model using the serotype data along with the locally reported rainfall could possibly capture the complicated dynamics better than the aggregate-serotype model. Although other climate variables (e.g., temperature) could be included, results from transfer entropy calculations indicated that they were not particularly relevant to dengue dynamics in these locations. As mentioned before, the Iquitos data were too sparse to use serotype data, but this could be attempted on the San Juan data, possibly with dimension reduction (e.g. Principal Component Analysis). Performance on the peak week could be possibly improved by putting time-of-year restrictions on the particular analogue sequences that are found. This would serve to look for analogues shapes, rather than actual case counts. The total cases predictions and peak height predictions would be less accurate; in this case two predictions could be made: one would be without restrictions, to predict total cases and the height of the peak; and the second would be with restrictions for peak location in time. All the models that are input to the ensemble typically predict total cases and the height of the peak better than they predict the time of the peak week, meaning that the individual methods were not sufficiently diversified. Generating a more diverse set of component models for the ensemble could also potentially be beneficial. The sparse peak week probability distributions could be improved by smoothing the distributions (especially in the early weeks of the season) to ensure that close predictions still score well.

## Supporting information

S1 File (ZIP)
