# Supplementary material for: Ensemble method for dengue prediction
Source: PLoS One. 2018 Jan 3;13(1):e0189988. doi: 10.1371/journal.pone.0189988 (PMC5752022; doi:10.1371/journal.pone.0189988)
Supplement: S1 File — (ZIP) [file pone.0189988.s001.zip › S1_File.pdf]

SUPPLEMENTAL INFORMATION for ***Ensemble method for dengue prediction*** by Anna L. Buczak, Benjamin Baugher, Linda J. Moniz, Thomas Bagley, Steven M. Babin, and Erhan Guven

This section describes the results of the individual models that were components of the ensemble model. The important points are that the individual models did not perform well and that their performance varied considerably from forecast to forecast. Please keep in mind that the data sets were not ideal and this was part of the Challenge. However, this also illustrates why the ensemble approach works better in this situation.

Examples of the Method of Analogues component model predictions for Iquitos and San Juan are shown in Figs A and B, respectively.

Fig A. Predictions for Iquitos, 2009. Model A used L= 2, V=4

Fig B. Results for San Juan, 2008. Model B used L=1,V=7

The accuracy for Iquitos can be seen in Table A for years 2009 to 2011.

Table A. Iquitos: Results of Predictions with respect to difference from the actual cases. Note that non-integer numbers are a result of averaging.

Prediction Year	Prediction Week	Difference in Peak Height from Ground Truth (# cases)	Difference in Peak Location from Ground Truth (week #)	Difference in Total Cases from Ground Truth
2009	0	13.6	18	23.8
2009	4	13.0	6	172.2
2009	8	8.4	10	4.0
2009	12	8.6	16	210.6
2009	16	2.6	4	24.0
2009	20	11.4	4	94.8
2009	24	12.8	17	31.8
2009	28	10.0	5	143.0
2009	32	9.2	8	100.0
2009	36	8.4	11	70.8
2009	40	7.8	15	79.4
2009	44	6.6	18	26.2
2009	48	7.6	18	19.2
2011	0	28.4	20	378.2
2011	4	14.4	8	368.0
2011	8	12.0	1	263.0
2011	12	7.6	0	159.2
2011	16	15.8	5	270.4
2011	20	6.4	11	91.2
2011	24	7.6	10	120.0
2011	28	1.2	14	36.8
2011	32	12.4	10	138.0
2011	36	1.0	6	12.4
2011	40	.4	15	33.0
2011	44	1.0	18	17.8
2011	48	.4	21	13.0

The Method of Analogues was much better at predicting the total number of cases in a dengue transmission season (particularly for Iquitos) than the week of the peak incidence.

Results for the Holt-Winters seasonal component model are shown in Figs C-J. Figs C and D show predictions for San Juan for periods of seasonality 52 and 104 (with all other parameters set to the same numbers: end of training: 797, optimization = RMSE). The prediction for the next 52 weeks is completely different depending on the period of seasonality.

Fig C. Holt-Winters prediction for San Juan, period =52, ending training data point = 797, RMSE optimization. The green vertical line shows the end of the training data, and the 53 predicted weeks end at the red line.

Fig D. Holt-Winters prediction for San Juan, period = 104, ending training data point = 797, RMSE optimization. The green vertical line shows the end of the training data, and the 53 predicted weeks end at the red line.

In Figs C and E, all parameters are exactly the same except for the ending week of the training data. In Fig C, it was week 797, while Fig E shows the predictions when the ending week is 820. The predictions are drastically different with high peaks being predicted when the last point of the training data is high (Fig C) and lower peaks being predicted when the last point of the training data is low.

Fig E. Holt-Winters prediction for San Juan, period = 52, ending training data point = 820, RMSE optimization. The green vertical line shows the end of the training data, and the 53 predicted weeks end at the red line.

Fig F shows predictions for San Juan for the period of seasonality week 52, end of training week 797, and optimization by MARE. The prediction is very similar to the one from Fig C where all the parameters were the same with the exception of the optimization method used (RMSE).

Fig F. Holt-Winters prediction for San Juan, period =52, ending training data point = 797, MARE optimization. The green vertical line shows the end of the training data, and the 53 predicted weeks end at the red line.

Figs G and H show predictions for Iquitos for periods of seasonality 53 and 103 (with all other parameters set to the same numbers: end of training: 400, optimization = RMSE). The prediction for the next 52 weeks is completely different depending on the period of seasonality.

Fig G. Holt-Winters prediction for Iquitos, period =53, ending training data point = 400, RMSE optimization. The green vertical line shows the end of the training data, and the 53 predicted weeks end at the red line.

Fig H. Holt-Winters prediction for Iquitos, period = 103, ending training data point = 400, RMSE optimization. The green vertical line shows the end of the training data, and the 53 predicted weeks end at the red line.

In Figs G and I, all parameters are exactly the same except for the ending week of the training data. In Fig G, it was 400, while Fig I shows the predictions when the ending week was 385. As for San Juan, the predictions are drastically different with high peaks being predicted when the last point of the training data is high (Fig G) and lower peaks being predicted when the last point of the training data is low (Fig I).

Fig I. Holt-Winters prediction for Iquitos, period = 53, ending training data point = 385, RMSE optimization. The green vertical line shows the end of the training data, and the 53 predicted weeks end at the red line.

Fig J shows predictions for Iquitos for the period of seasonality 53, end of training 400, and optimization by MARE. The prediction is very similar to the one from Fig G where all the parameters were the same with the exception of the optimization method used (RMSE).

Fig J. Holt-Winters prediction for Iquitos, period = 53, ending training data point = 400, using MARE optimization. The green vertical line shows the end of the training data. The red vertical line shows the end of the 53 predicted weeks.

Historical model results are shown in Figs K-P. Recall from the text that these histograms were computed from the training data (for Iquitos, 2000 – 2009; for San Juan, 1990- 2009) for each of the three variables. The San Juan histograms are shown in Figs K-M, while the Iquitos histograms in shown in Figs N-P. In cases where the histogram has one maximum bin (Figs K, M, N), the middle of this bin will be always predicted by the historical model (e.g., 28 for peak week for San Juan). In cases where there is more than one maximum bin (Figs L, O, P), each of the middle bin values is predicted with a probability 1/count (i.e., 0.5 probability for peak value for San Juan). The historical model for the total number of cases for Iquitos is an especially poor model, as there are 8 bins with the same height (height of 1). This means that this model will predict one of those bins with a probability of 0.125. This illustrates the great difficulty of making predictions for Iquitos.

Fig K. Histogram of Peak Week for San Juan.

Fig L. Histogram of Peak Value for San Juan.

Fig M. Histogram of Total cases in a Transmission Season for San Juan.

Fig N. Histogram of Peak Week for Iquitos.

Fig O. Histogram of Peak Value for Iquitos.

Fig P. Histogram of Total cases in a Transmission Season for Iquitos.

Finally, we now present figures illustrating how the 300 chosen individual component models performed compared to the ensemble model that resulted from using these 300 component models. Figs Q-BB are for Iquitos, while Figs CC-NN are for San Juan. The abscissa is always the week in which a prediction was made. For each of the four dengue transmission seasons in Iquitos, Figs Q-T are for peak height (i.e., maximum number of weekly cases), Figs U-X are for peak week (i.e., week in which peak height occurred), and Figs Y-BB are for the total dengue cases for each transmission season. For each of the four dengue transmission seasons in San Juan, Figs CC-FF are for peak height, Figs GG-JJ are for peak week, and Figs KK-NN are for total dengue cases for each transmission season. The performance of the component models varied widely, even for cases in which the Holt-Winters type of component model dominated. The ensemble predictions became more accurate as more data for a given season became available.

Fig Q. Plot of ensemble model performance for peak height compared to the performance of the individual component models making up that ensemble model. These results are for the first dengue season for Iquitos.

Fig R. Plot of ensemble model performance for peak height compared to the performance of the individual component models making up that ensemble model. These results are for the second dengue season for Iquitos.

Fig S. Plot of ensemble model performance for peak height compared to the performance of the individual component models making up that ensemble model. These results are for the third dengue season for Iquitos.

Fig T. Plot of ensemble model performance for peak height compared to the performance of the individual component models making up that ensemble model. These results are for the fourth dengue season for Iquitos.

Fig U. Plot of ensemble model performance for peak week compared to the performance of the individual component models making up that ensemble model. These results are for the first dengue season for Iquitos.

Fig V. Plot of ensemble model performance for peak week compared to the performance of the individual component models making up that ensemble model. These results are for the second dengue season for Iquitos.

Fig W. Plot of ensemble model performance for peak week compared to the performance of the individual component models making up that ensemble model. These results are for the third dengue season for Iquitos.

Fig X. Plot of ensemble model performance for peak week compared to the performance of the individual component models making up that ensemble model. These results are for the fourth dengue season for Iquitos.

Fig Y. Plot of ensemble model performance for total dengue season cases compared to the performance of the individual component models making up that ensemble model. These results are for the first dengue season for Iquitos.

Fig Z. Plot of ensemble model performance for total dengue season cases compared to the performance of the individual component models making up that ensemble model. These results are for the second dengue season for Iquitos.

Fig AA. Plot of ensemble model performance for total dengue season cases compared to the performance of the individual component models making up that ensemble model. These results are for the third dengue season for Iquitos.

Fig BB. Plot of ensemble model performance for total dengue season cases compared to the performance of the individual component models making up that ensemble model. These results are for the fourth dengue season for Iquitos.

Fig CC. Plot of ensemble model performance for peak height compared to the performance of the individual component models making up that ensemble model. These results are for the first dengue season for San Juan.

Fig DD. Plot of ensemble model performance for peak height compared to the performance of the individual component models making up that ensemble model. These results are for the second dengue season for San Juan.

Fig EE. Plot of ensemble model performance for peak height compared to the performance of the individual component models making up that ensemble model. These results are for the third dengue season for San Juan.

Fig FF. Plot of ensemble model performance for peak height compared to the performance of the individual component models making up that ensemble model. These results are for the fourth dengue season for San Juan.

Fig GG. Plot of ensemble model performance for peak week compared to the performance of the individual component models making up that ensemble model. These results are for the first dengue season for San Juan.

Fig HH. Plot of ensemble model performance for peak week compared to the performance of the individual component models making up that ensemble model. These results are for the second dengue season for San Juan.

Fig II. Plot of ensemble model performance for peak week compared to the performance of the individual component models making up that ensemble model. These results are for the third dengue season for San Juan.

Fig JJ. Plot of ensemble model performance for peak week compared to the performance of the individual component models making up that ensemble model. These results are for the fourth dengue season for San Juan.

Fig KK. Plot of ensemble model performance for total dengue season cases compared to the performance of the individual component models making up that ensemble model. These results are for the first dengue season for San Juan.

Fig LL. Plot of ensemble model performance for total dengue season cases compared to the performance of the individual component models making up that ensemble model. These results are for the second dengue season for San Juan.

Fig MM. Plot of ensemble model performance for total dengue season cases compared to the performance of the individual component models making up that ensemble model. These results are for the third dengue season for San Juan.

Fig NN. Plot of ensemble model performance for total dengue season cases compared to the performance of the individual component models making up that ensemble model. These results are for the fourth dengue season for San Juan.
