# Supplementary figures and images for: Ensemble method for dengue prediction
Source: PLoS One. 2018 Jan 3;13(1):e0189988. doi: 10.1371/journal.pone.0189988 (PMC5752022; doi:10.1371/journal.pone.0189988)

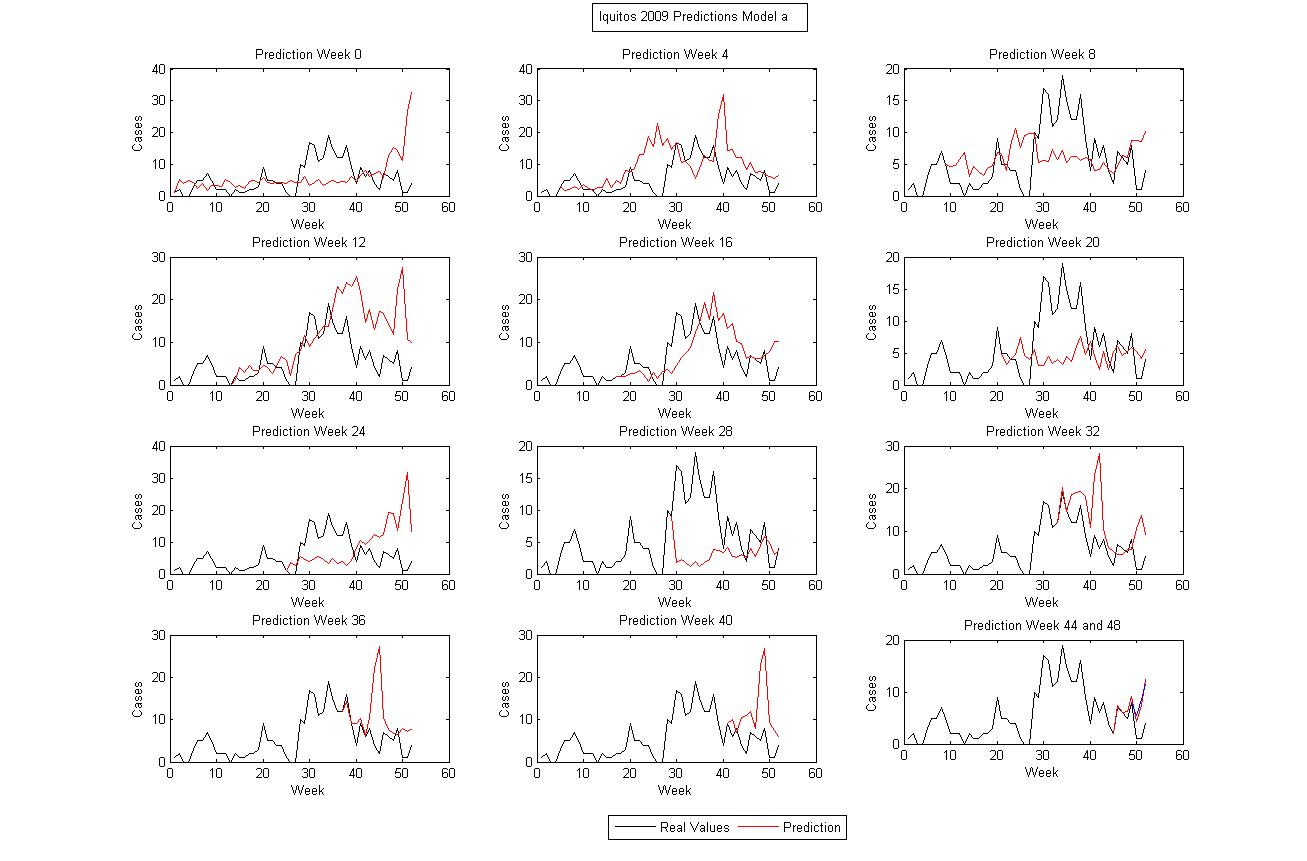

Supplement: S1 File — (ZIP) [file pone.0189988.s001.zip › S1_FigA.tif]

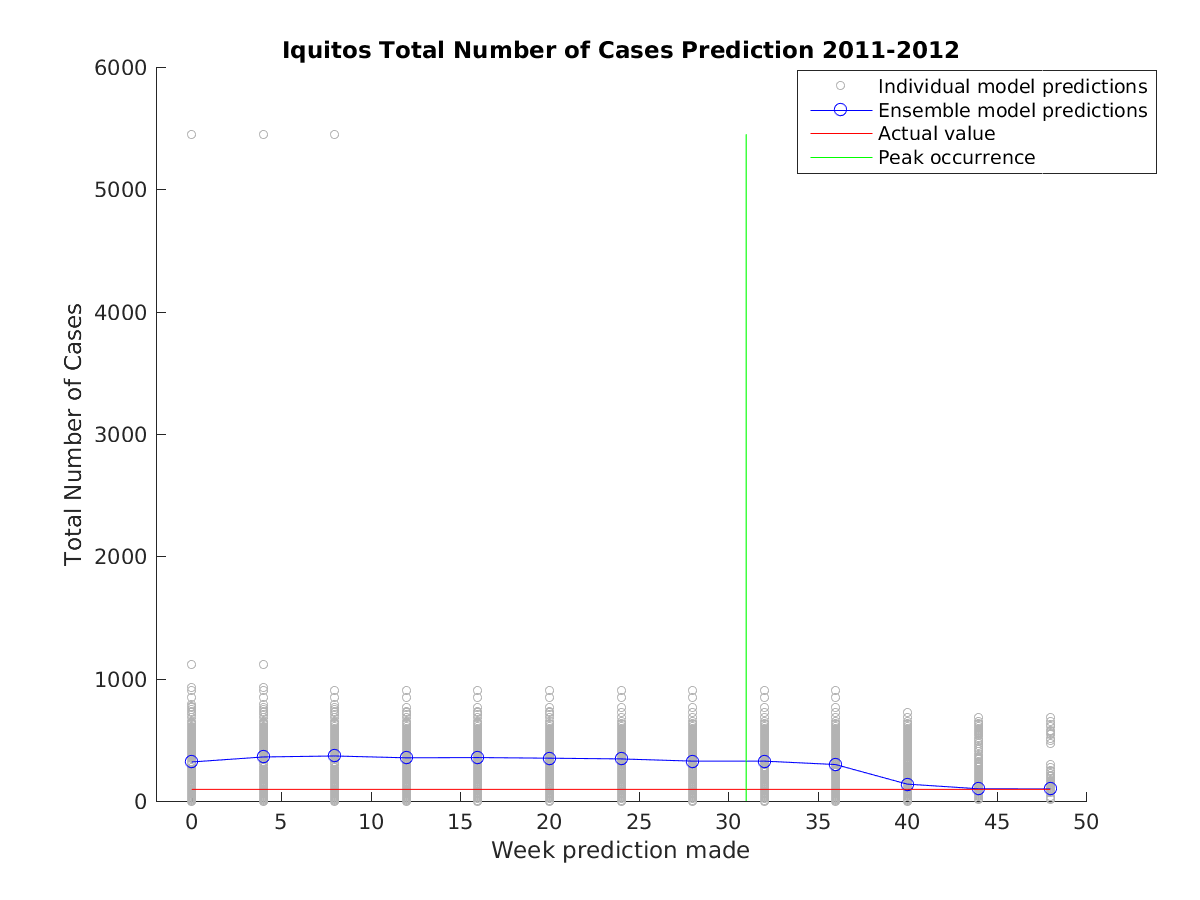

Supplement: S1 File — (ZIP) [file pone.0189988.s001.zip › S1_FigAA.tif]

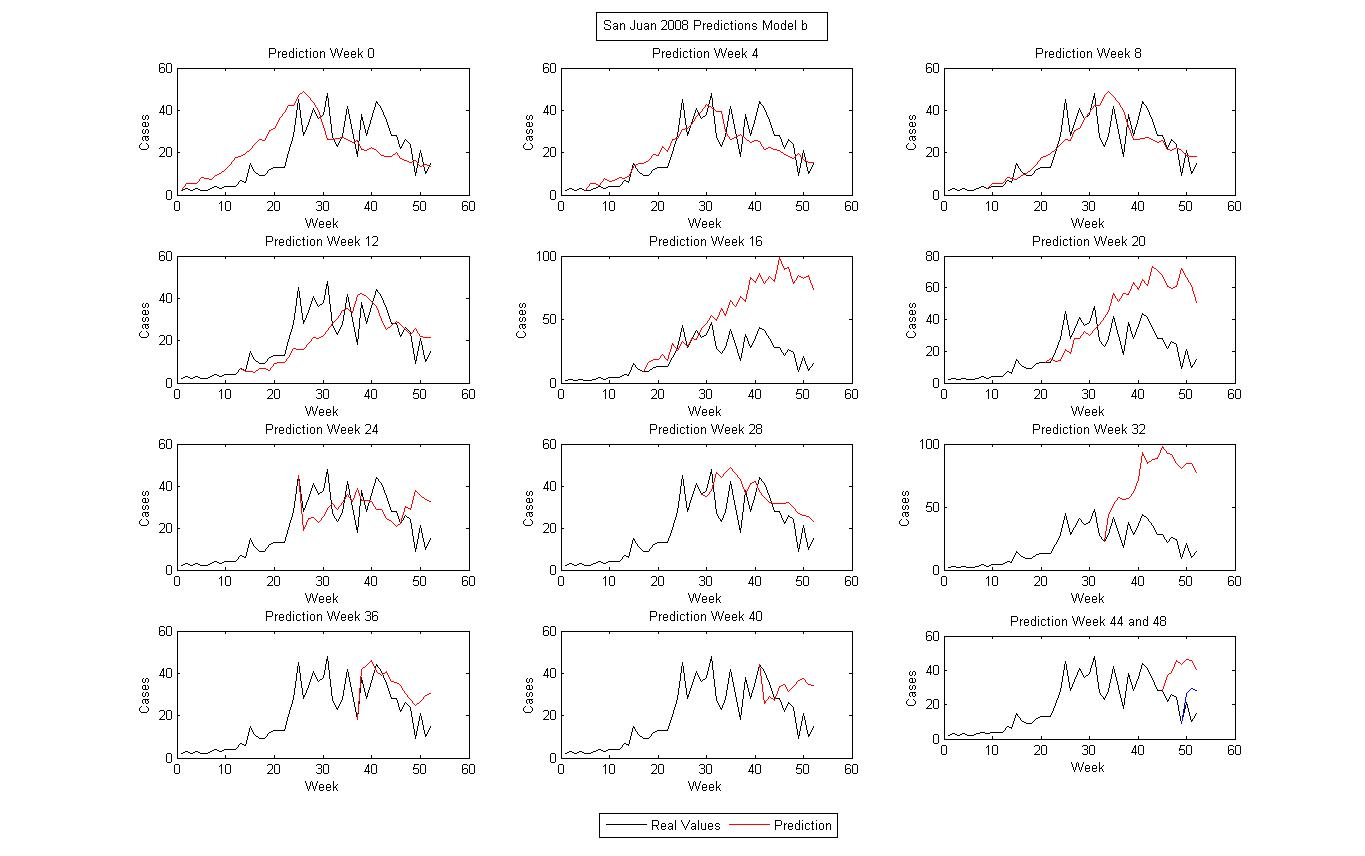

Supplement: S1 File — (ZIP) [file pone.0189988.s001.zip › S1_FigB.tif]

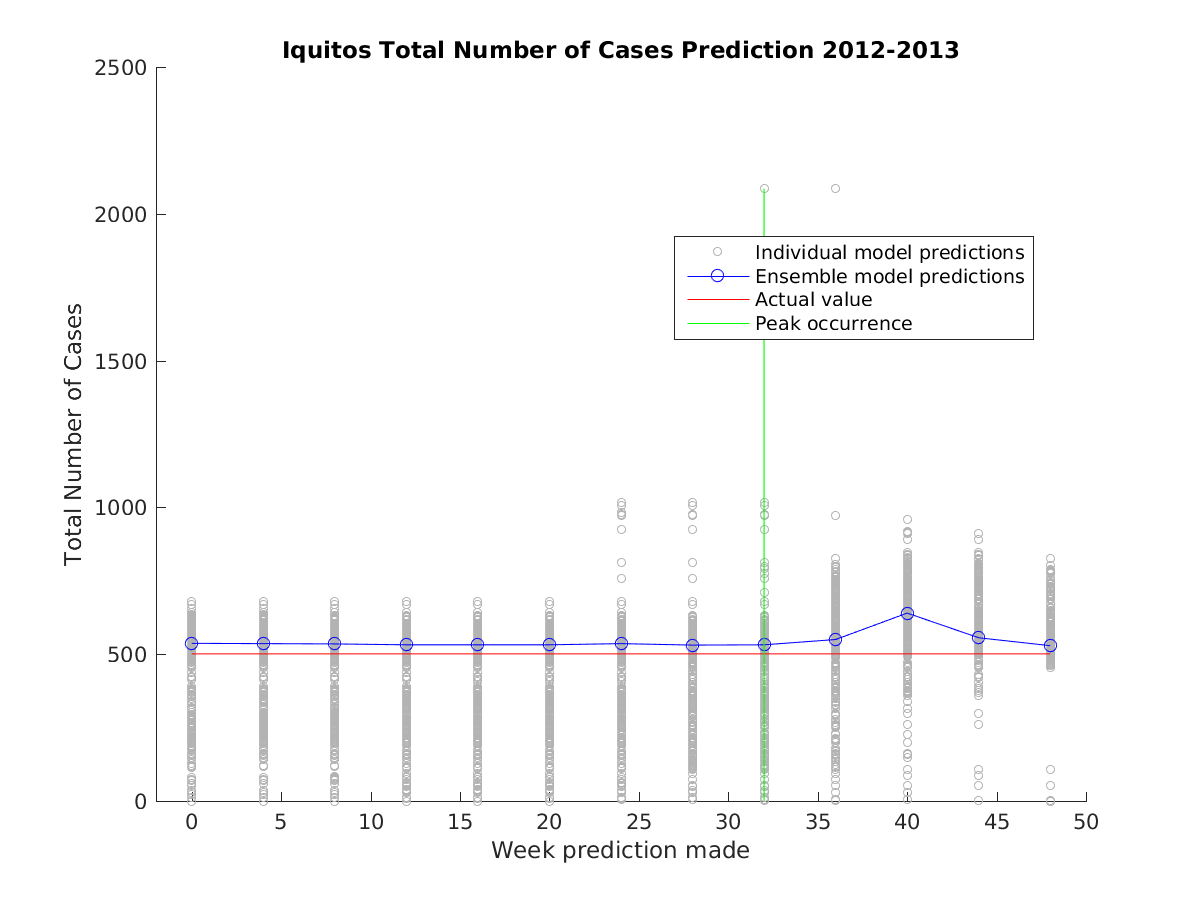

Supplement: S1 File — (ZIP) [file pone.0189988.s001.zip › S1_FigBB.tif]

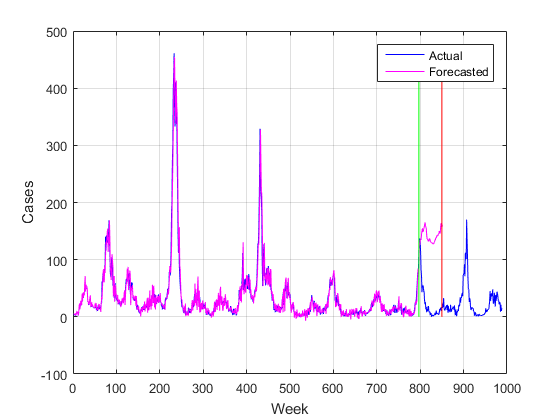

Supplement: S1 File — (ZIP) [file pone.0189988.s001.zip › S1_FigC.tif]

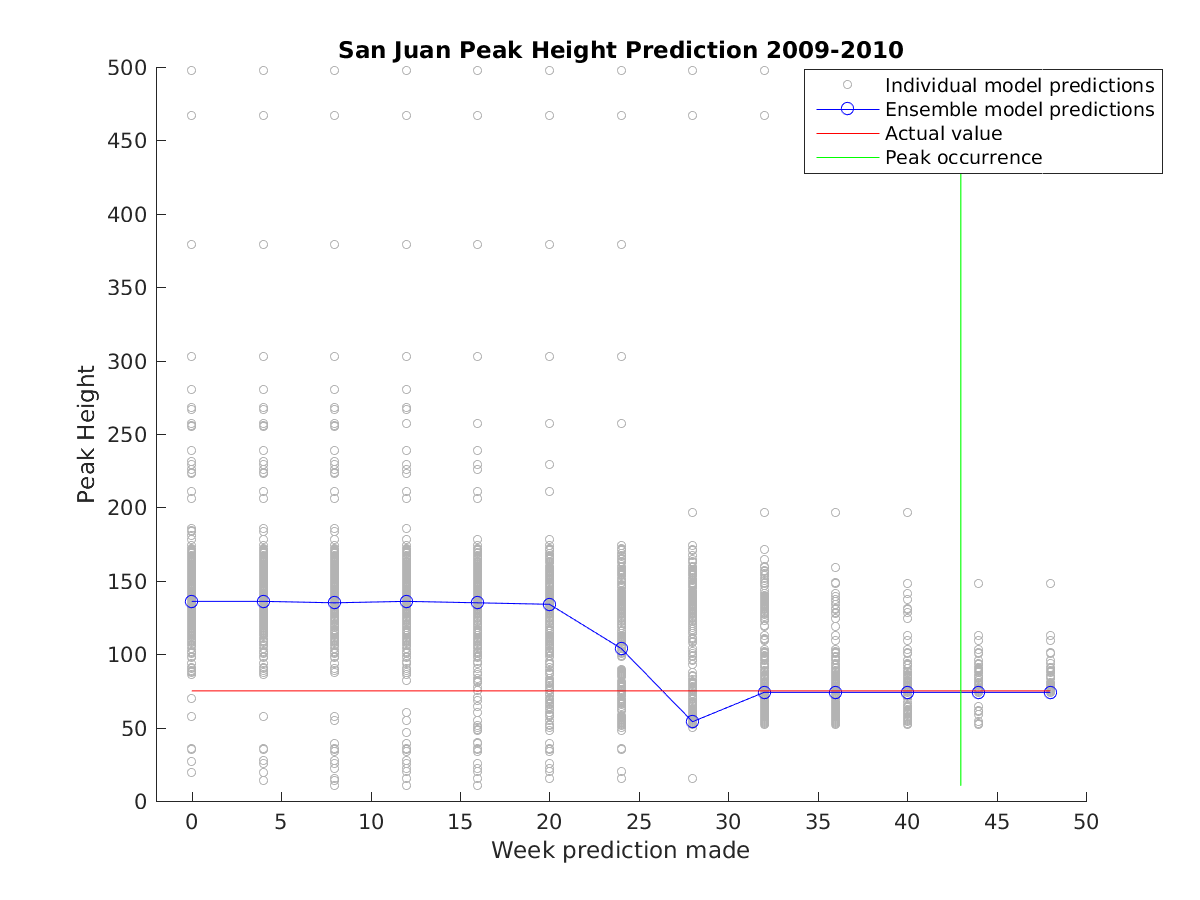

Supplement: S1 File — (ZIP) [file pone.0189988.s001.zip › S1_FigCC.tif]

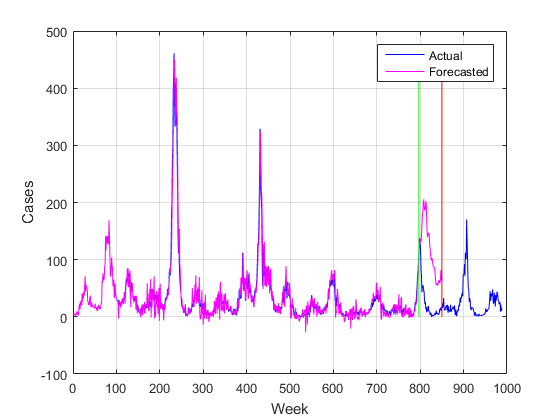

Supplement: S1 File — (ZIP) [file pone.0189988.s001.zip › S1_FigD.tif]

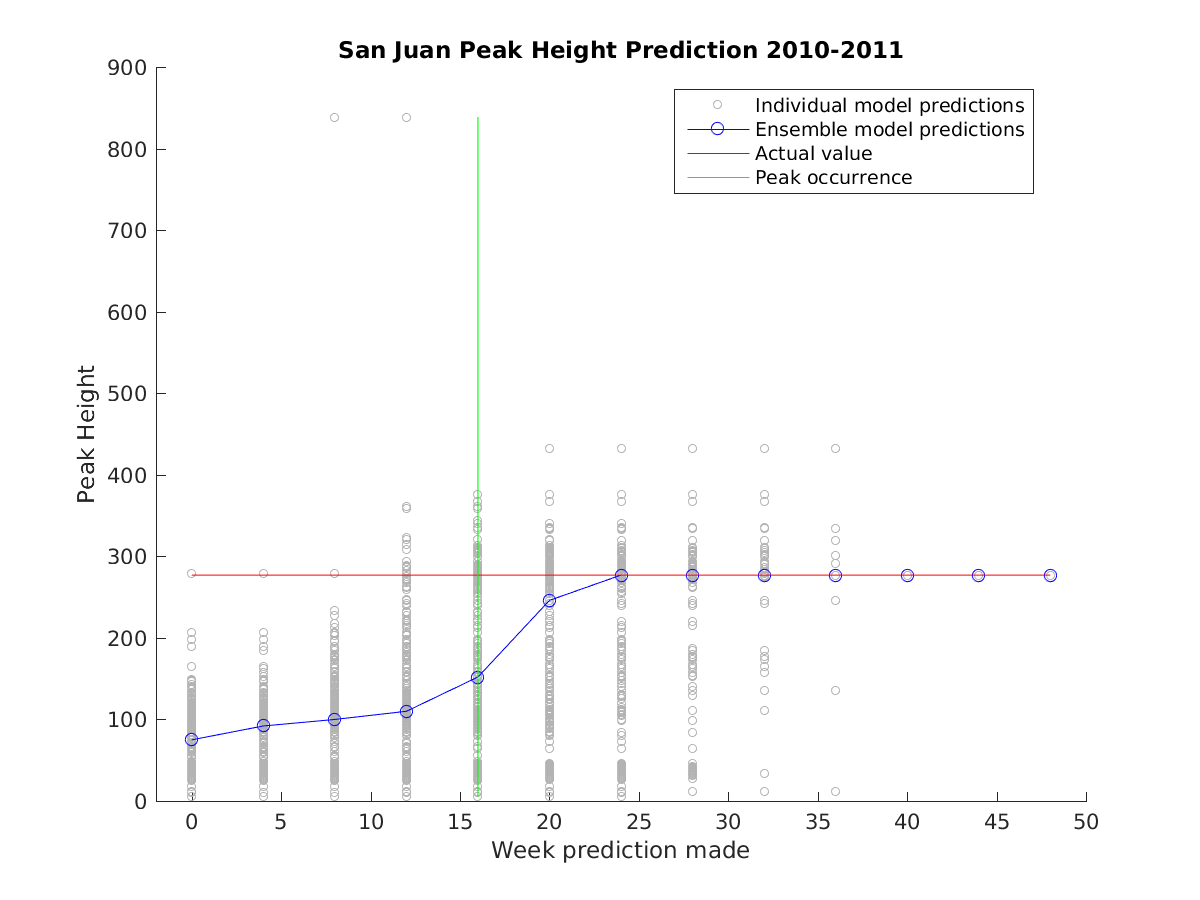

Supplement: S1 File — (ZIP) [file pone.0189988.s001.zip › S1_FigDD.tif]

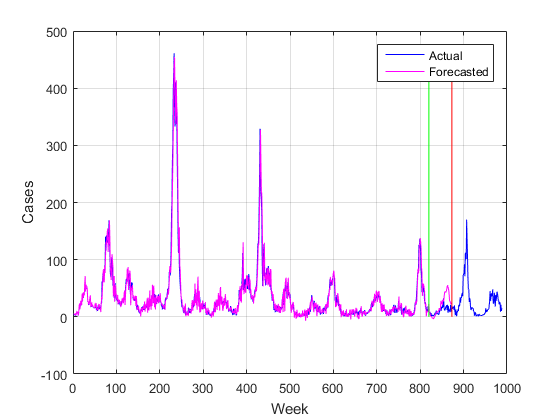

Supplement: S1 File — (ZIP) [file pone.0189988.s001.zip › S1_FigE.tif]

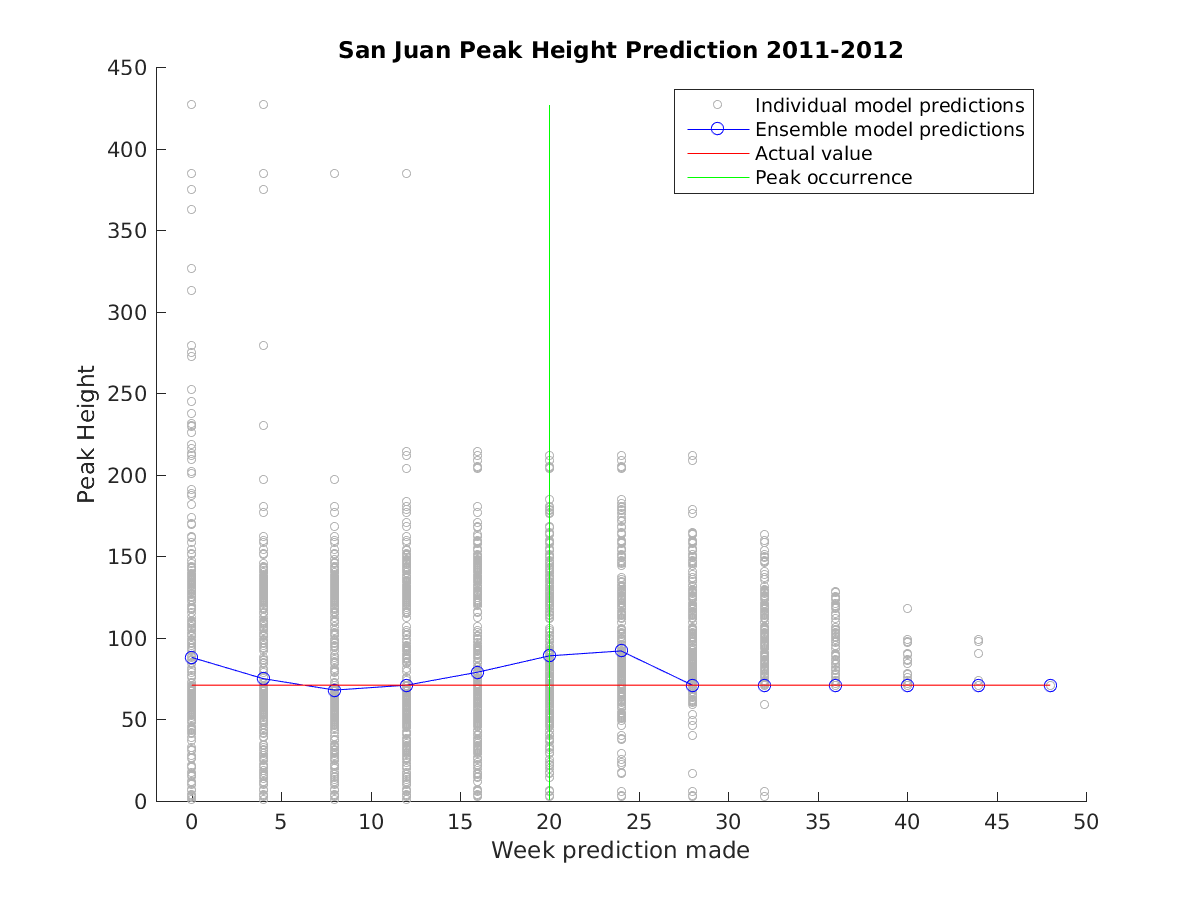

Supplement: S1 File — (ZIP) [file pone.0189988.s001.zip › S1_FigEE.tif]

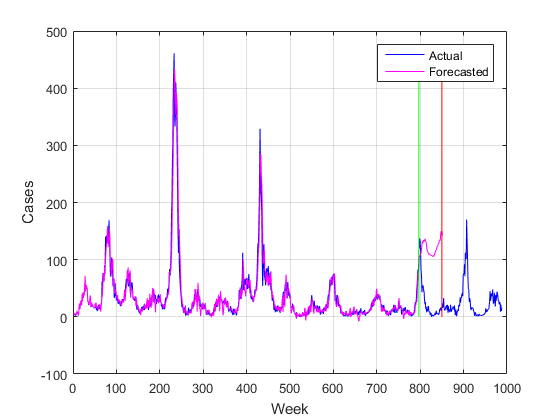

Supplement: S1 File — (ZIP) [file pone.0189988.s001.zip › S1_FigF.tif]

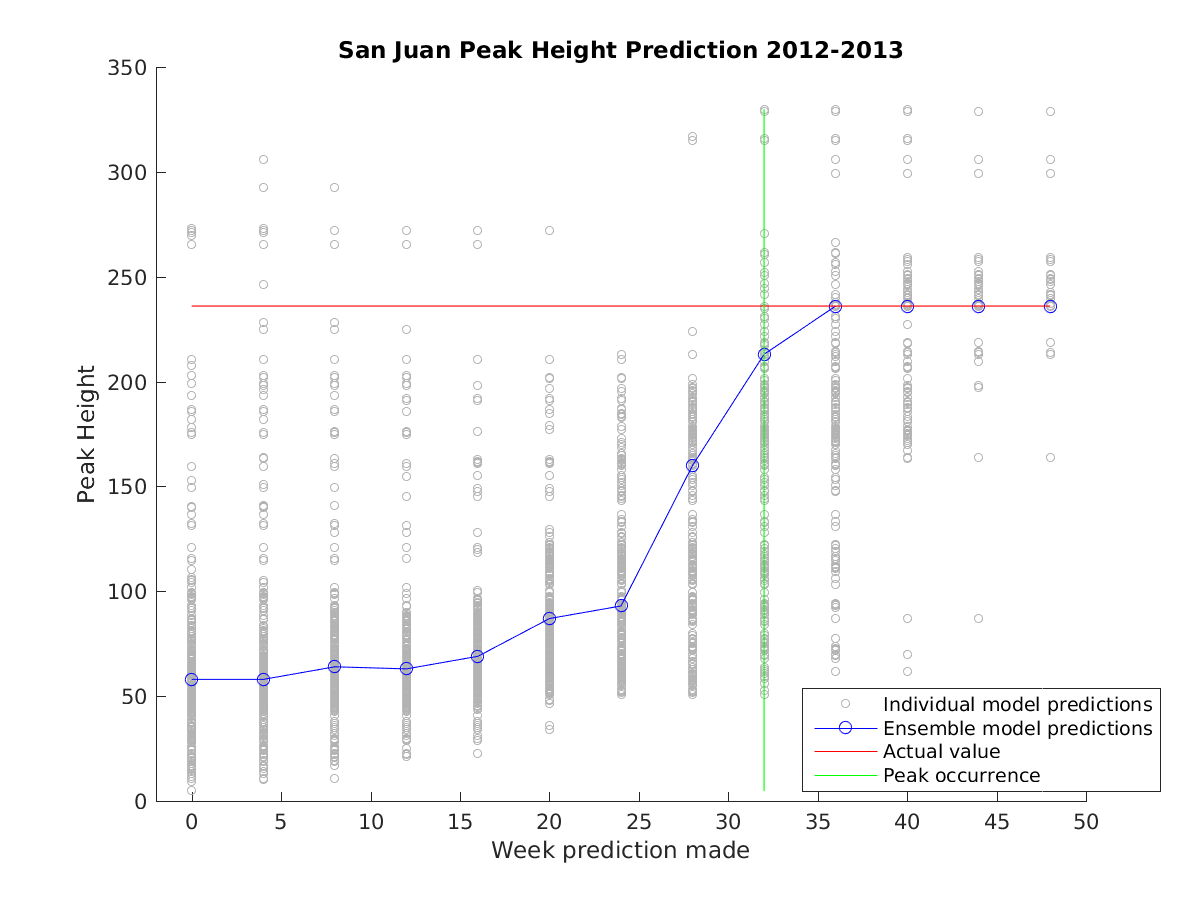

Supplement: S1 File — (ZIP) [file pone.0189988.s001.zip › S1_FigFF.tif]

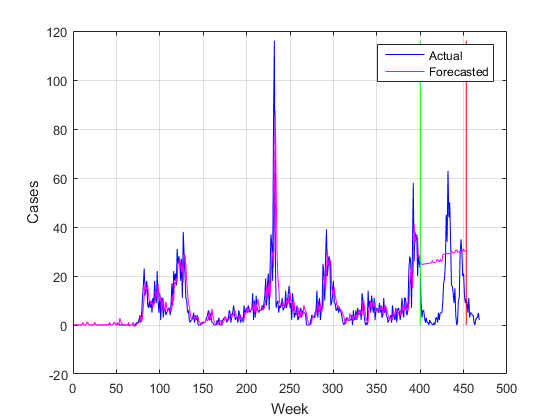

Supplement: S1 File — (ZIP) [file pone.0189988.s001.zip › S1_FigG.tif]

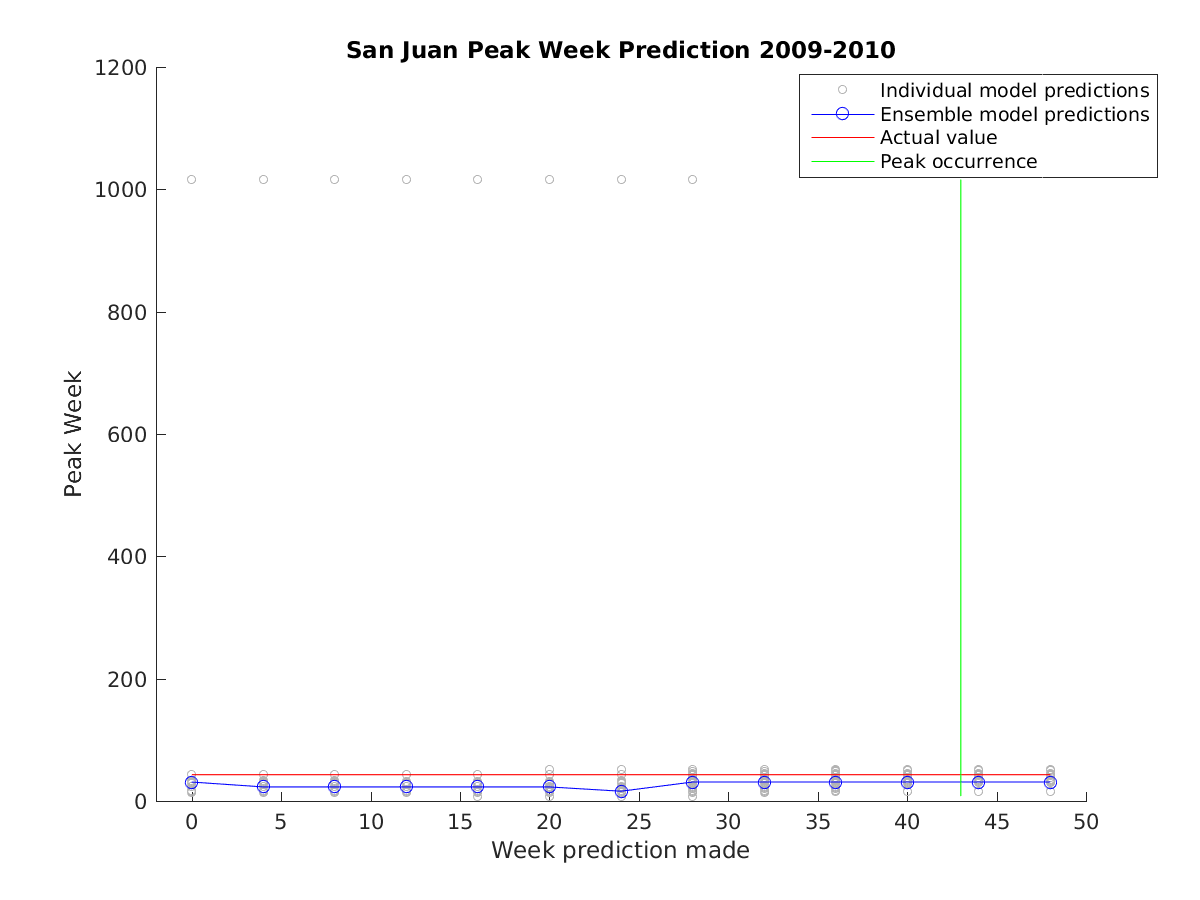

Supplement: S1 File — (ZIP) [file pone.0189988.s001.zip › S1_FigGG.tif]

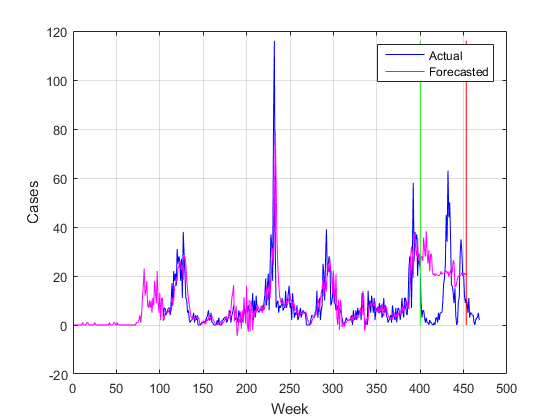

Supplement: S1 File — (ZIP) [file pone.0189988.s001.zip › S1_FigH.tif]

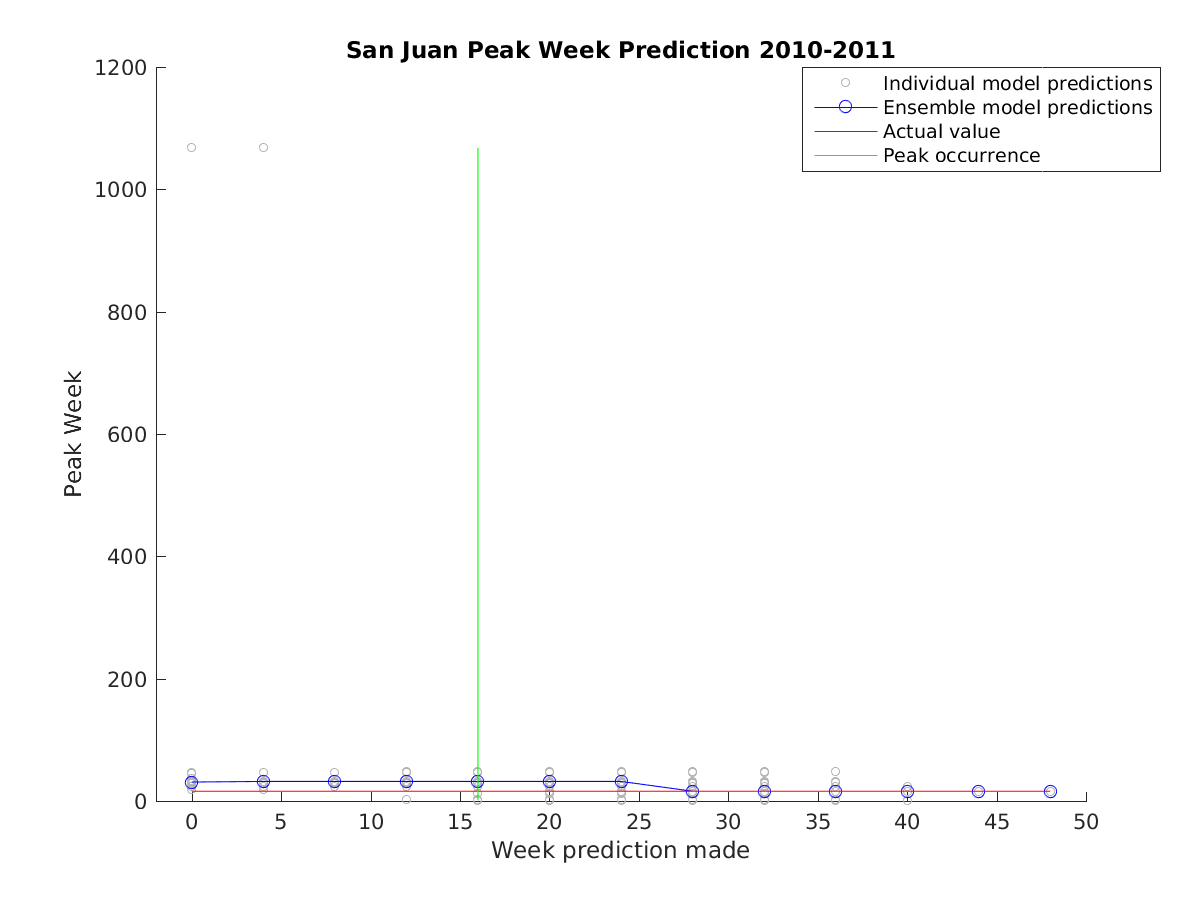

Supplement: S1 File — (ZIP) [file pone.0189988.s001.zip › S1_FigHH.tif]

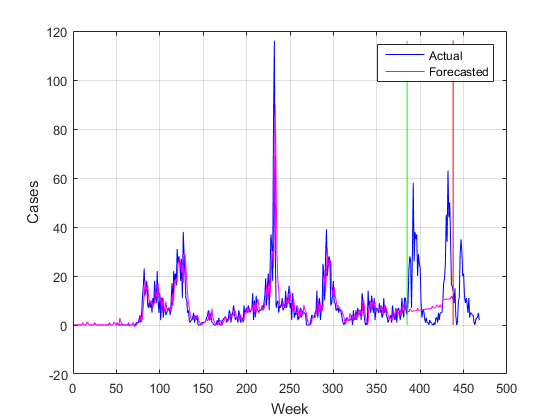

Supplement: S1 File — (ZIP) [file pone.0189988.s001.zip › S1_FigI.tif]

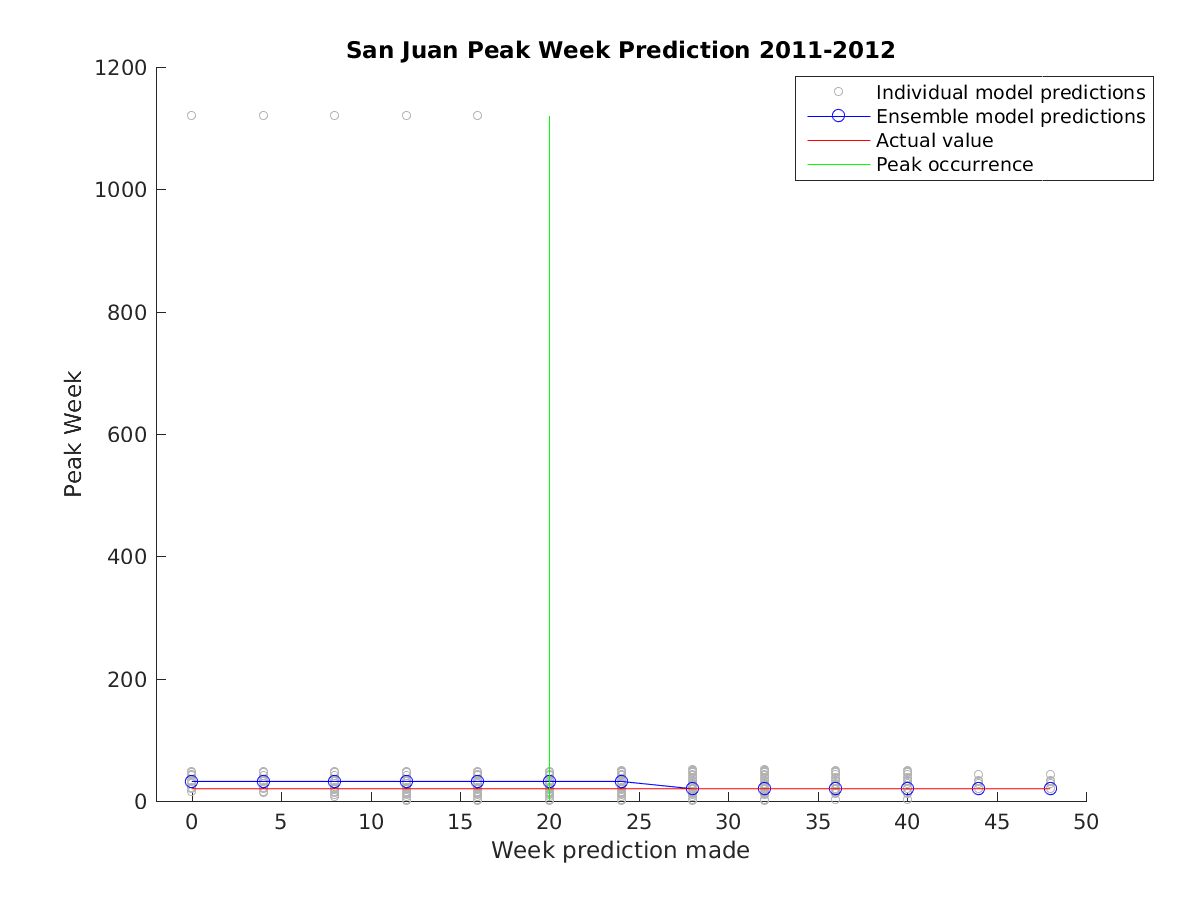

Supplement: S1 File — (ZIP) [file pone.0189988.s001.zip › S1_FigII.tif]

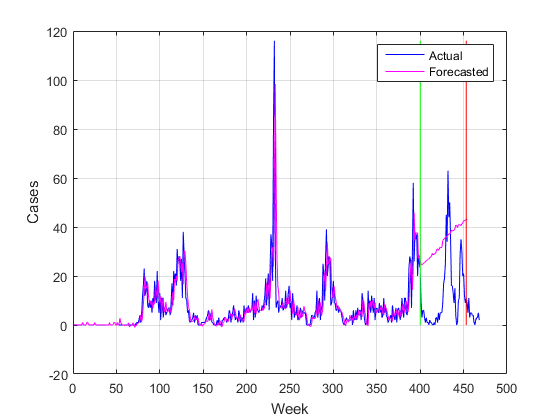

Supplement: S1 File — (ZIP) [file pone.0189988.s001.zip › S1_FigJ.tif]

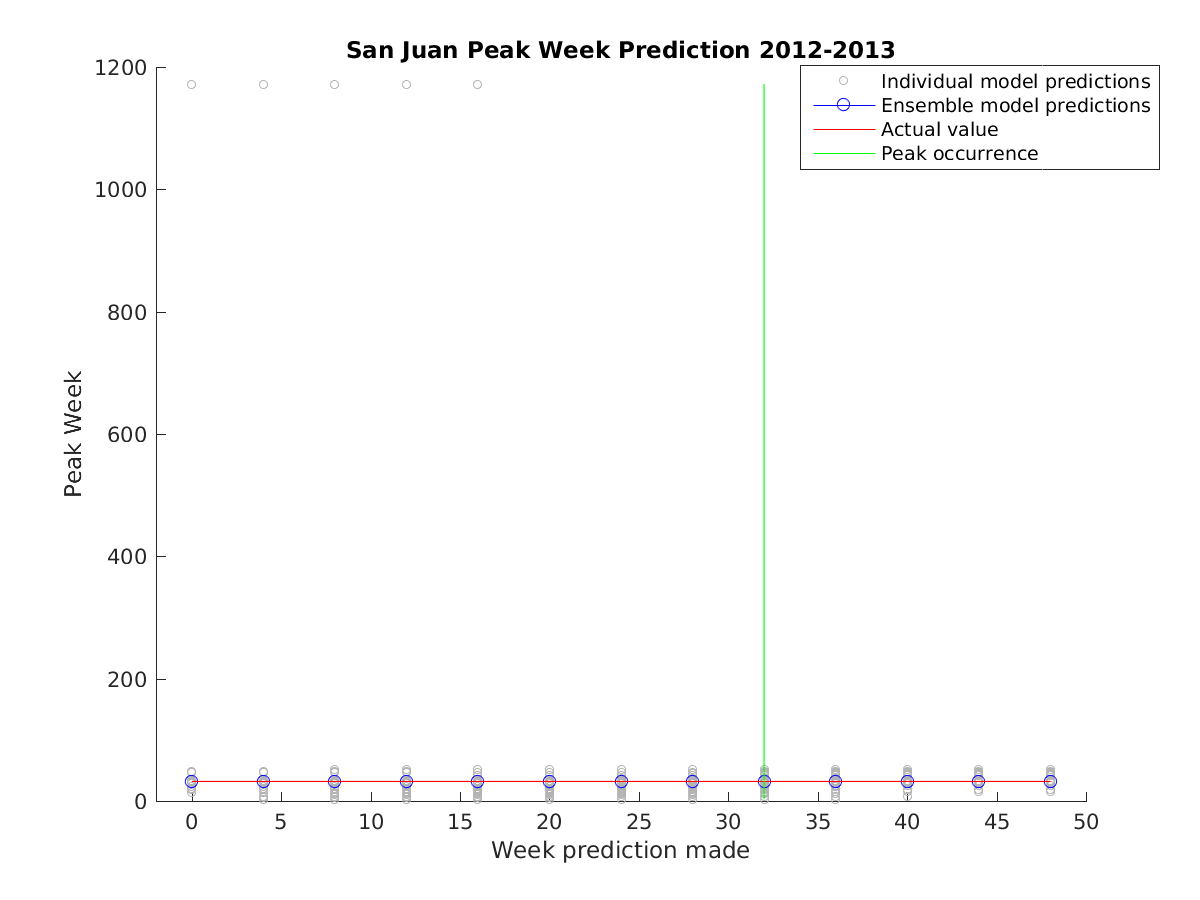

Supplement: S1 File — (ZIP) [file pone.0189988.s001.zip › S1_FigJJ.tif]

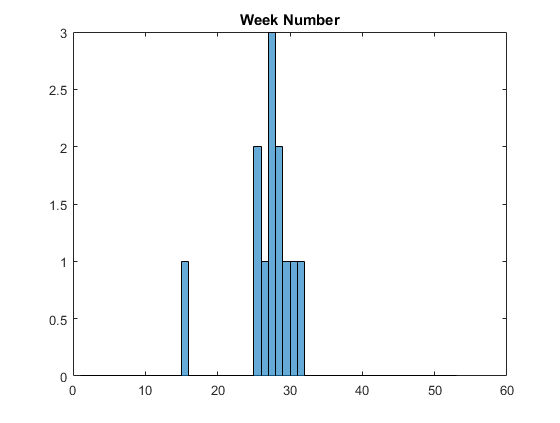

Supplement: S1 File — (ZIP) [file pone.0189988.s001.zip › S1_FigK.tif]

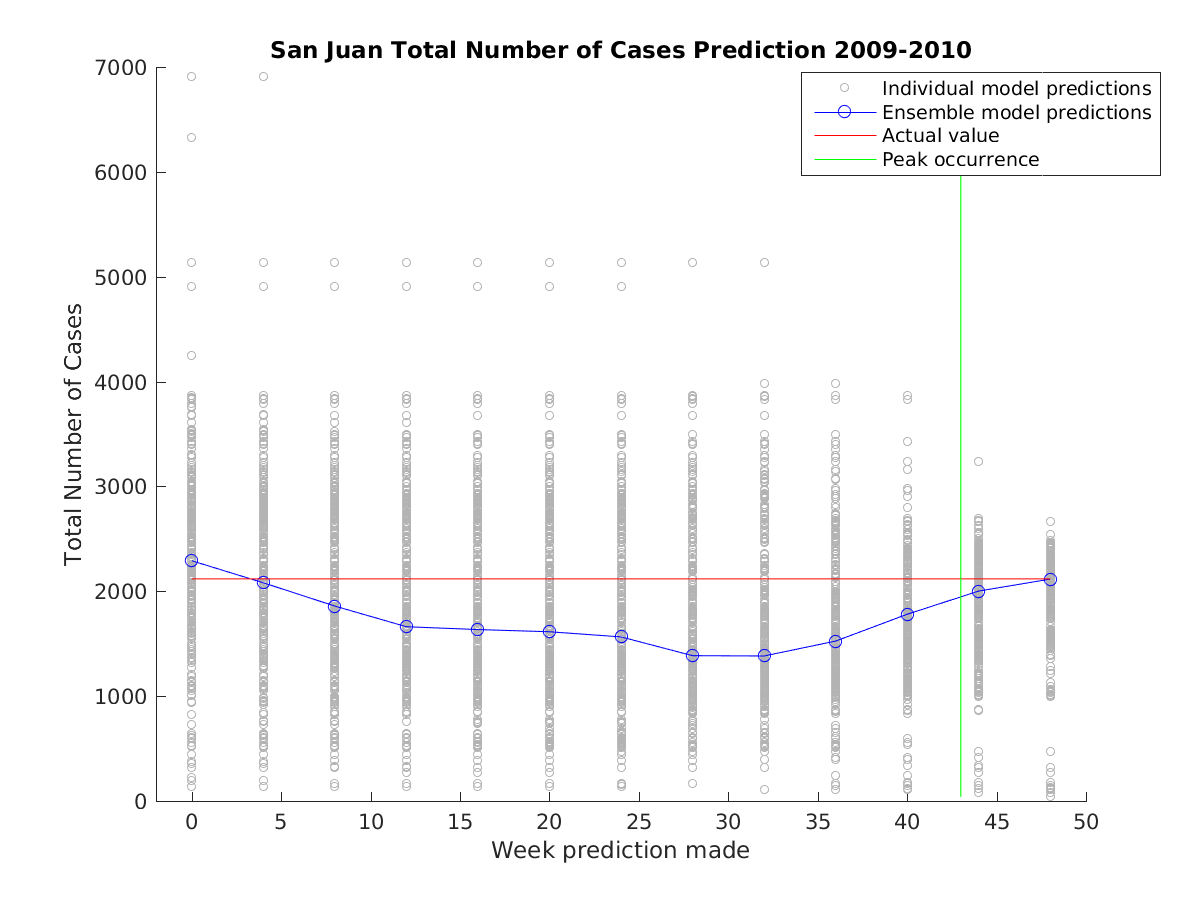

Supplement: S1 File — (ZIP) [file pone.0189988.s001.zip › S1_FigKK.tif]

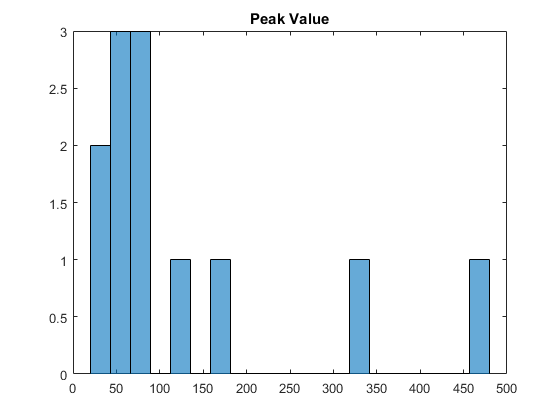

Supplement: S1 File — (ZIP) [file pone.0189988.s001.zip › S1_FigL.tif]

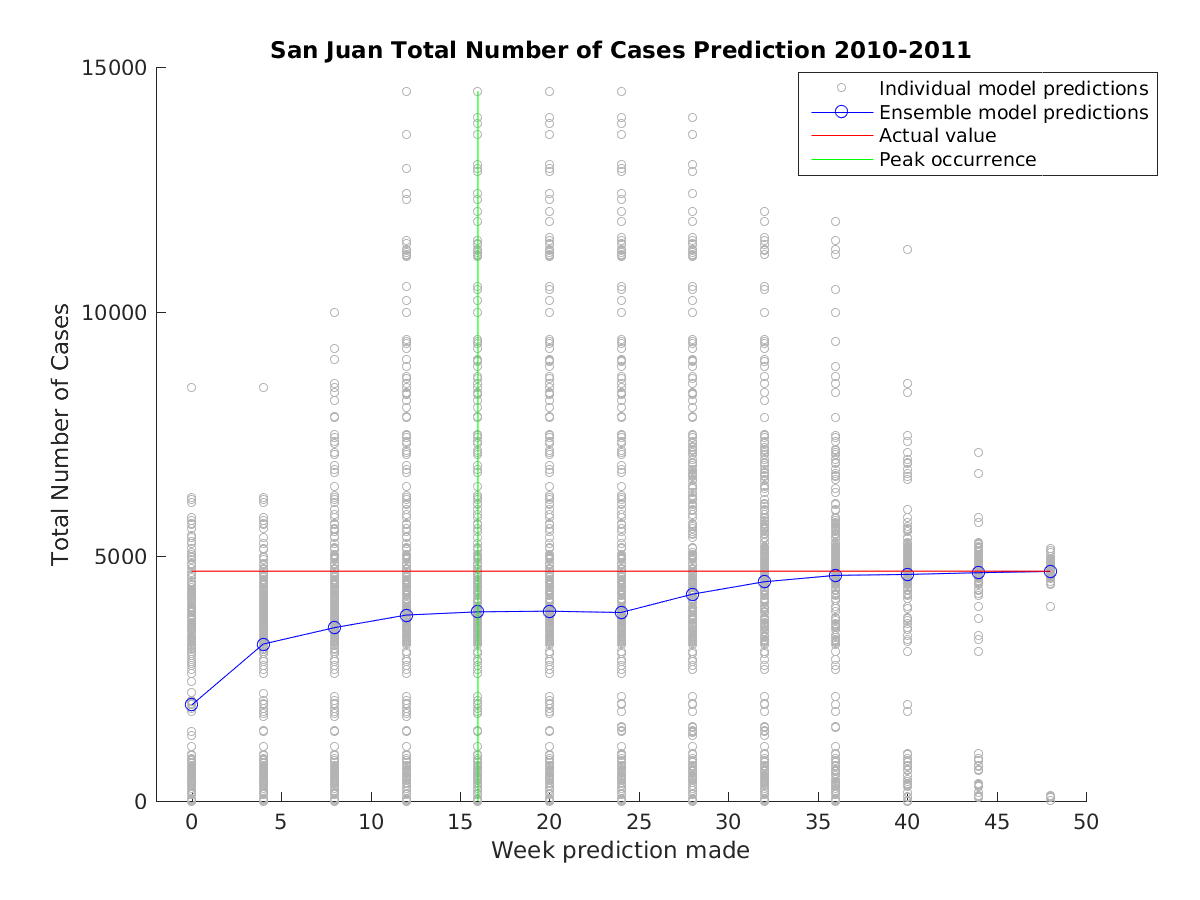

Supplement: S1 File — (ZIP) [file pone.0189988.s001.zip › S1_FigLL.tif]

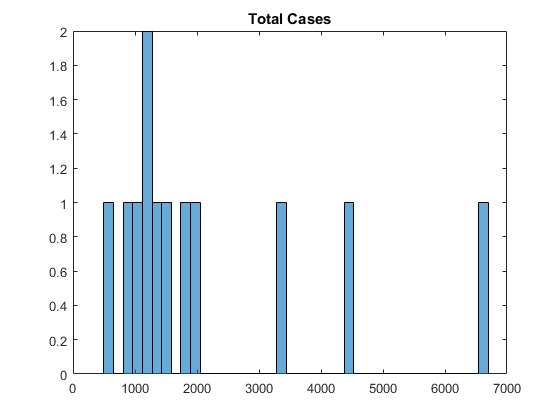

Supplement: S1 File — (ZIP) [file pone.0189988.s001.zip › S1_FigM.tif]

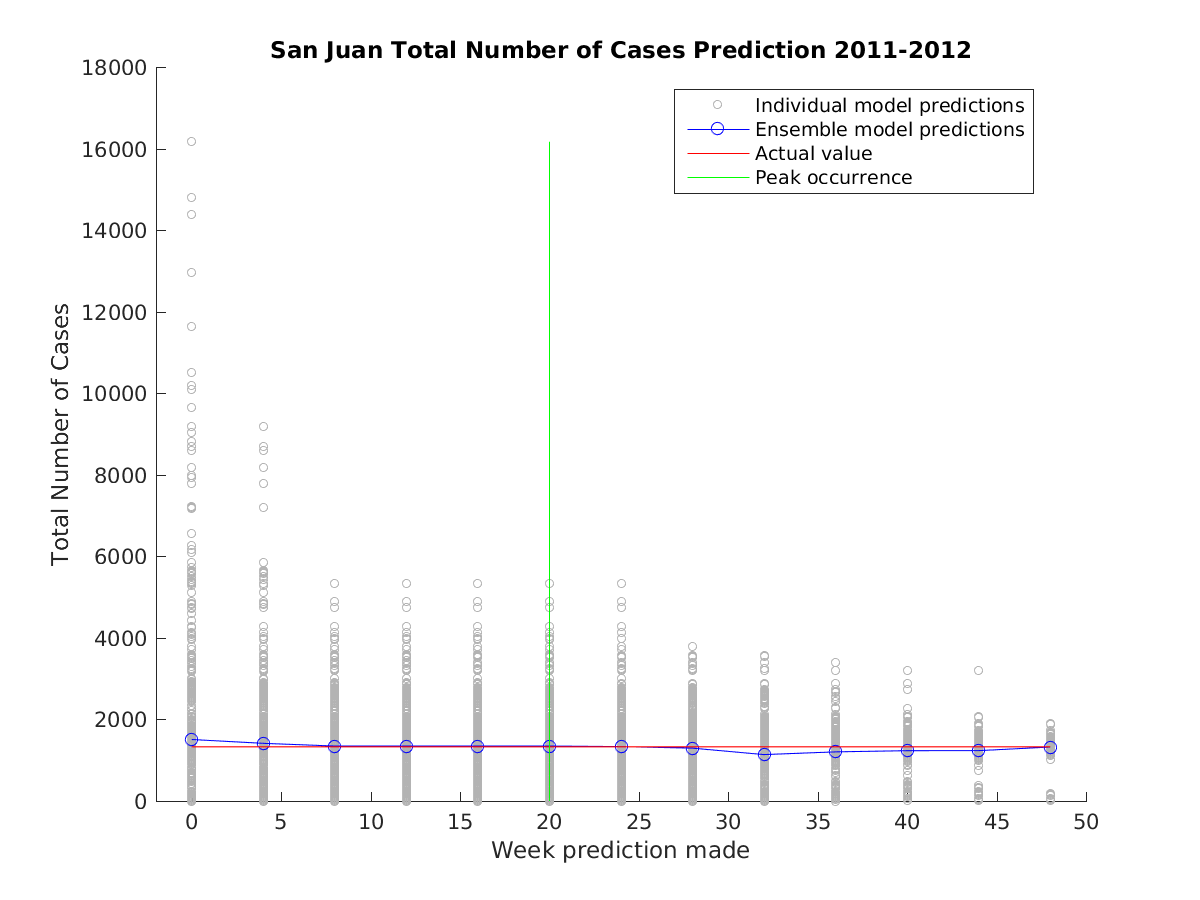

Supplement: S1 File — (ZIP) [file pone.0189988.s001.zip › S1_FigMM.tif]

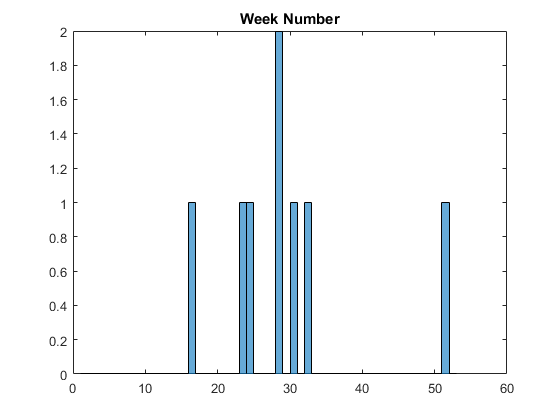

Supplement: S1 File — (ZIP) [file pone.0189988.s001.zip › S1_FigN.tif]

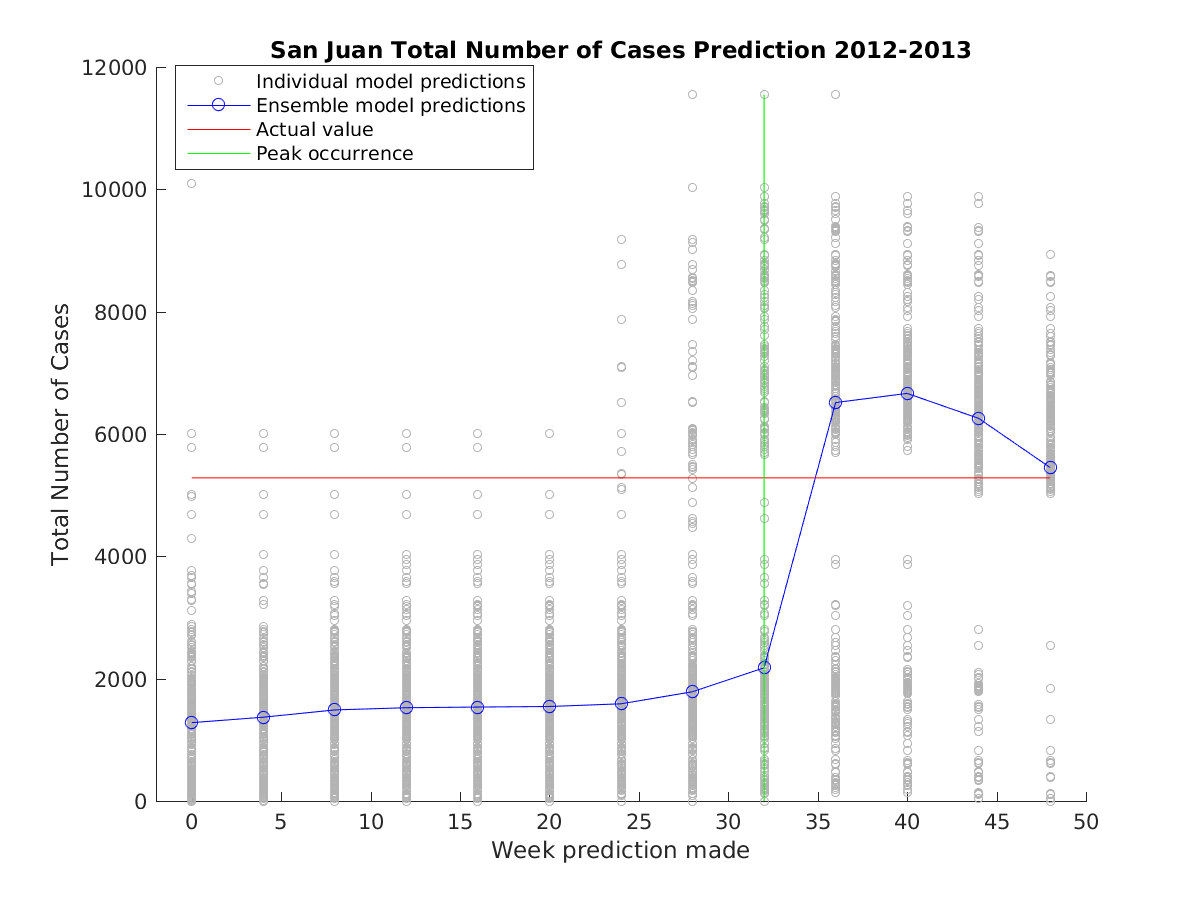

Supplement: S1 File — (ZIP) [file pone.0189988.s001.zip › S1_FigNN.tif]

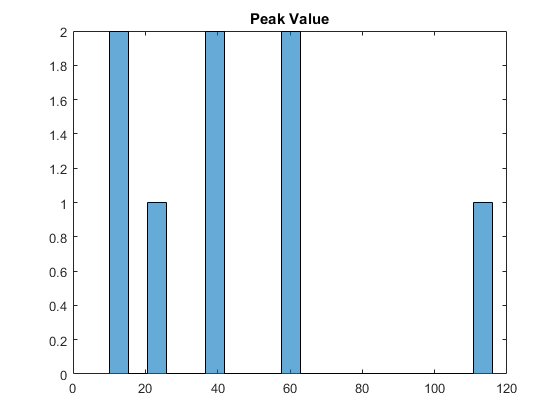

Supplement: S1 File — (ZIP) [file pone.0189988.s001.zip › S1_FigO.tif]

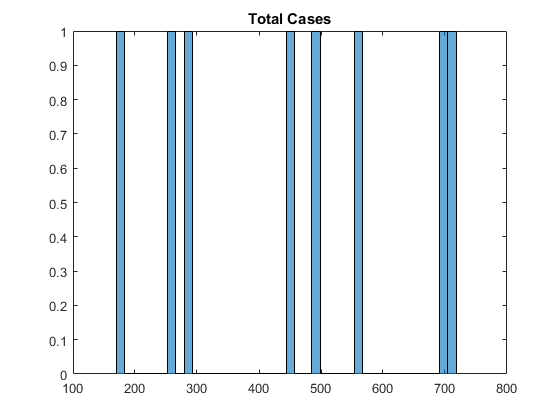

Supplement: S1 File — (ZIP) [file pone.0189988.s001.zip › S1_FigP.tif]

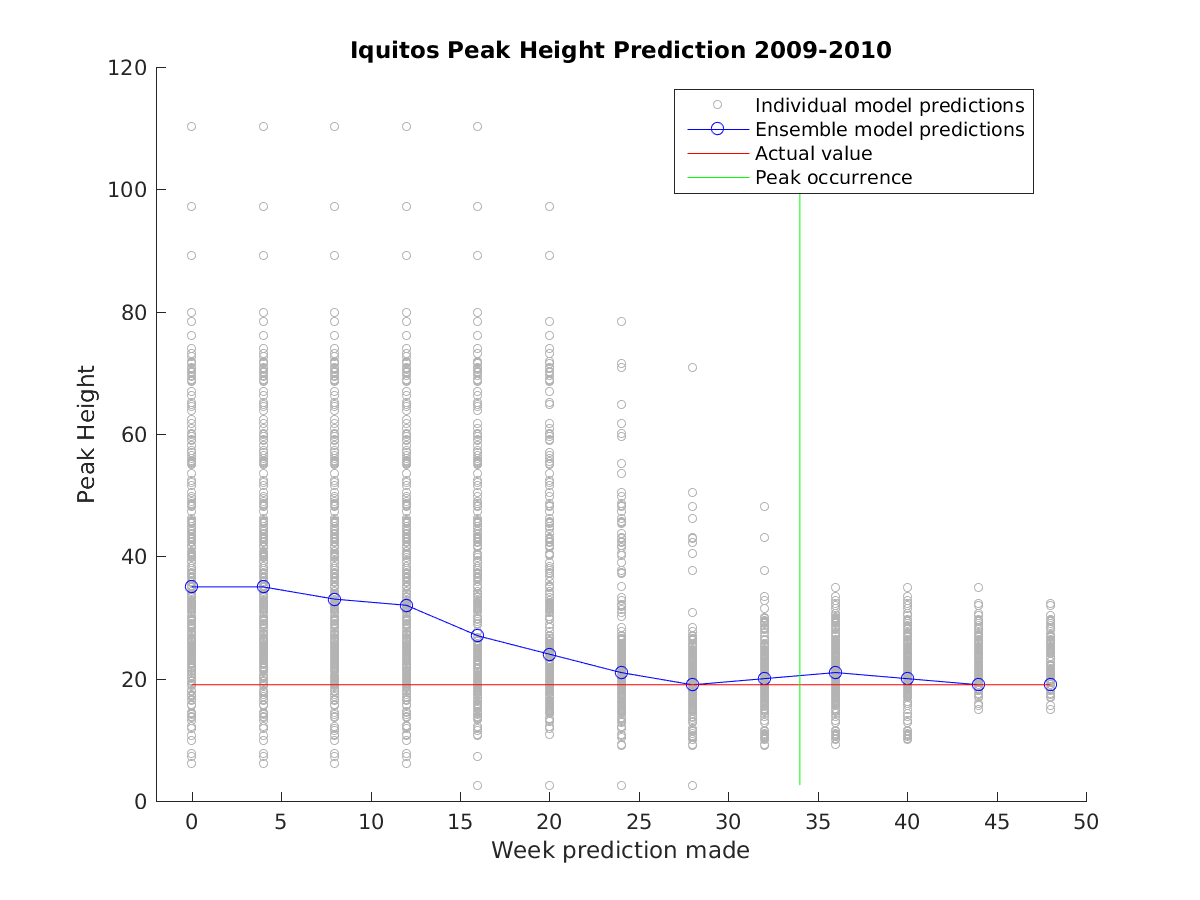

Supplement: S1 File — (ZIP) [file pone.0189988.s001.zip › S1_FigQ.tif]

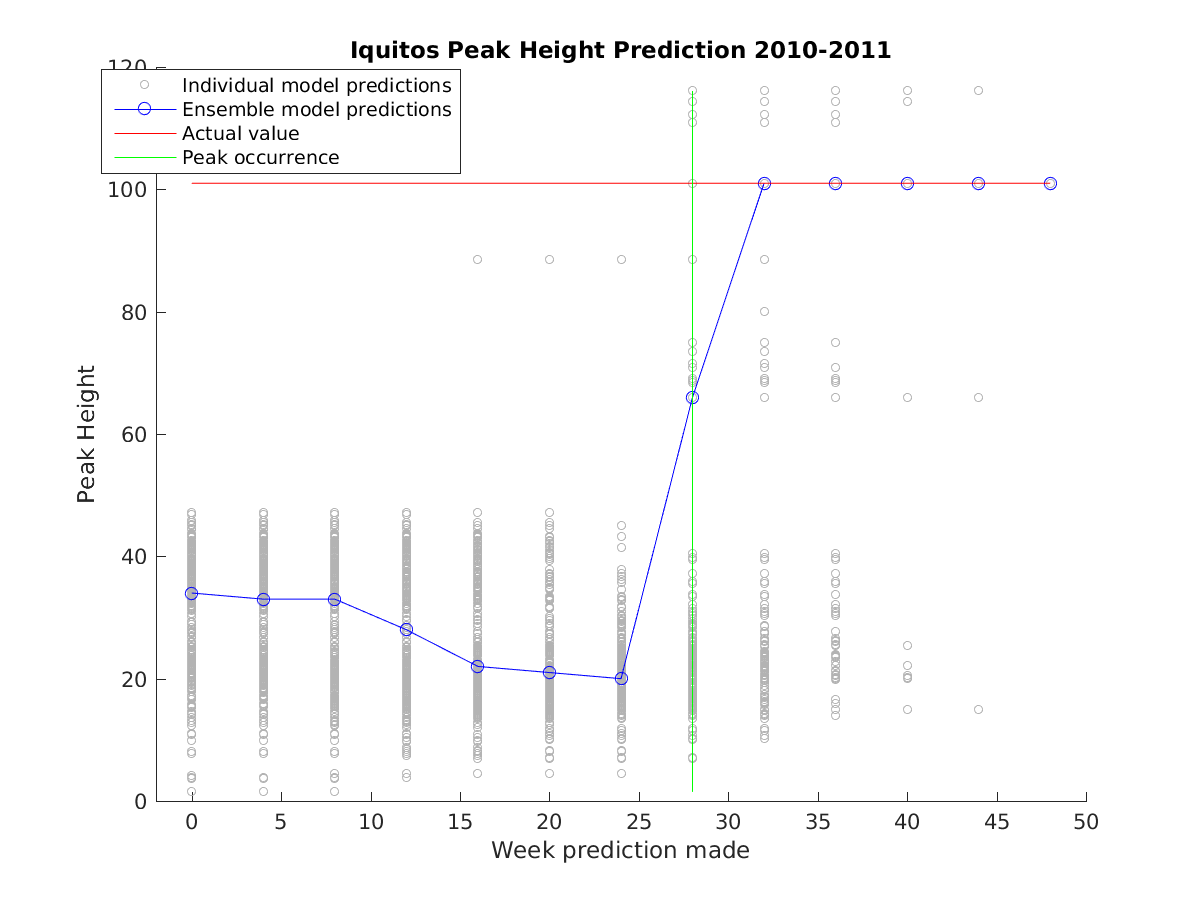

Supplement: S1 File — (ZIP) [file pone.0189988.s001.zip › S1_FigR.tif]

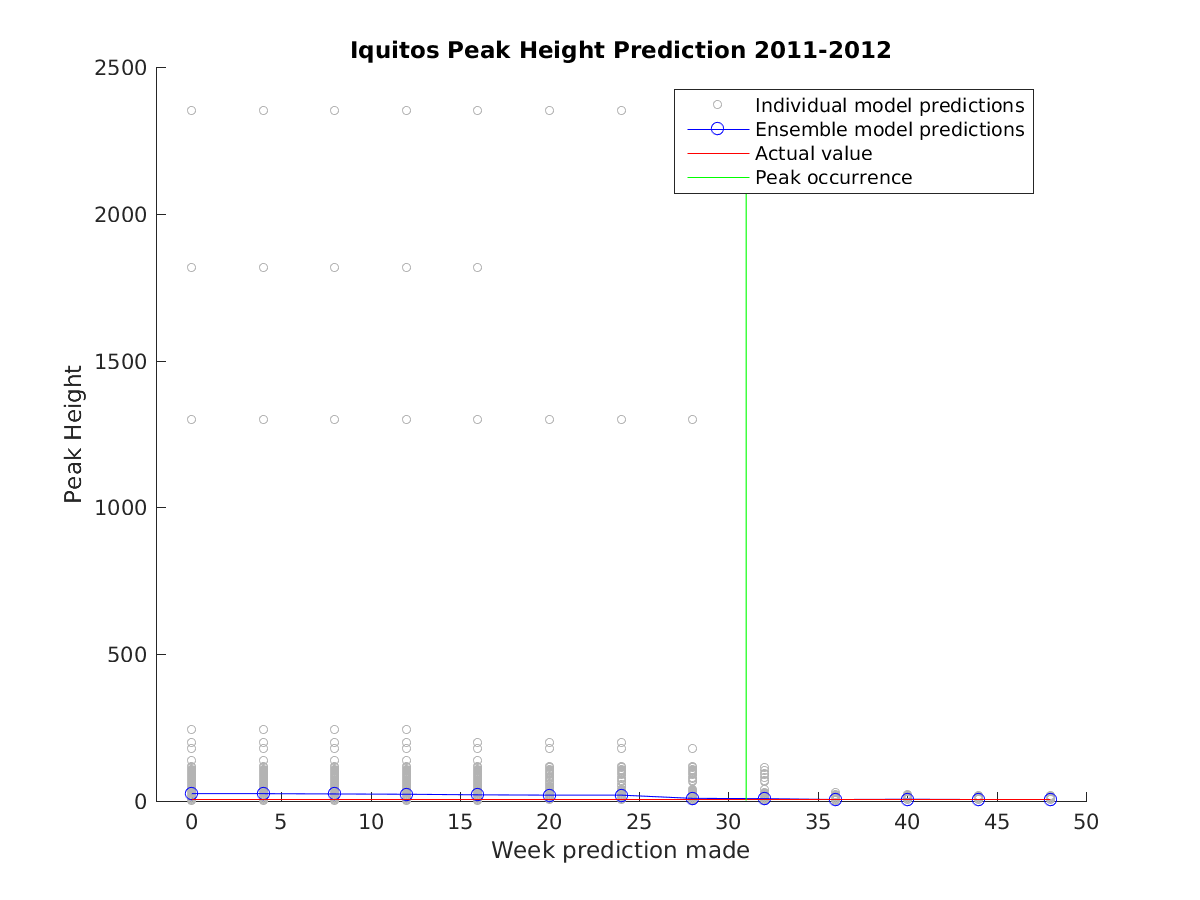

Supplement: S1 File — (ZIP) [file pone.0189988.s001.zip › S1_FigS.tif]

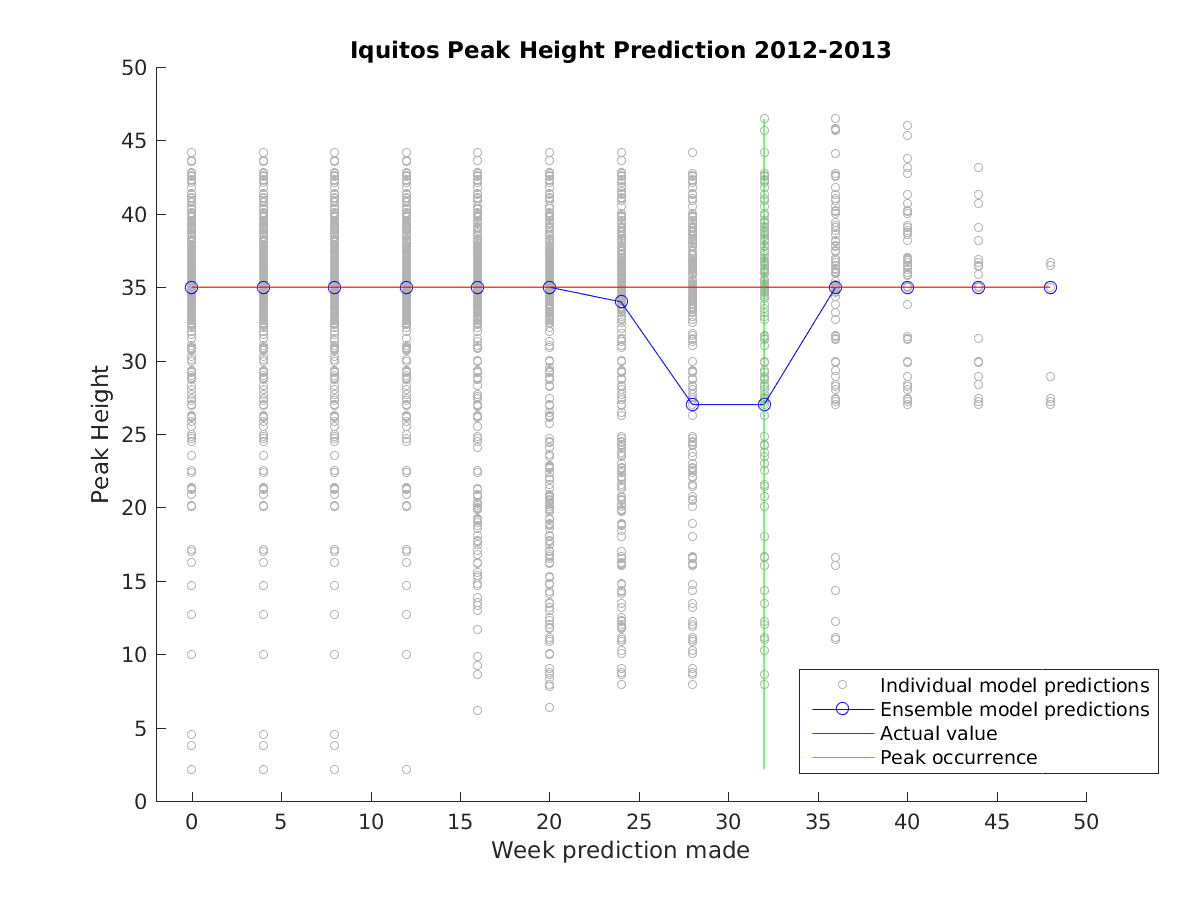

Supplement: S1 File — (ZIP) [file pone.0189988.s001.zip › S1_FigT.tif]

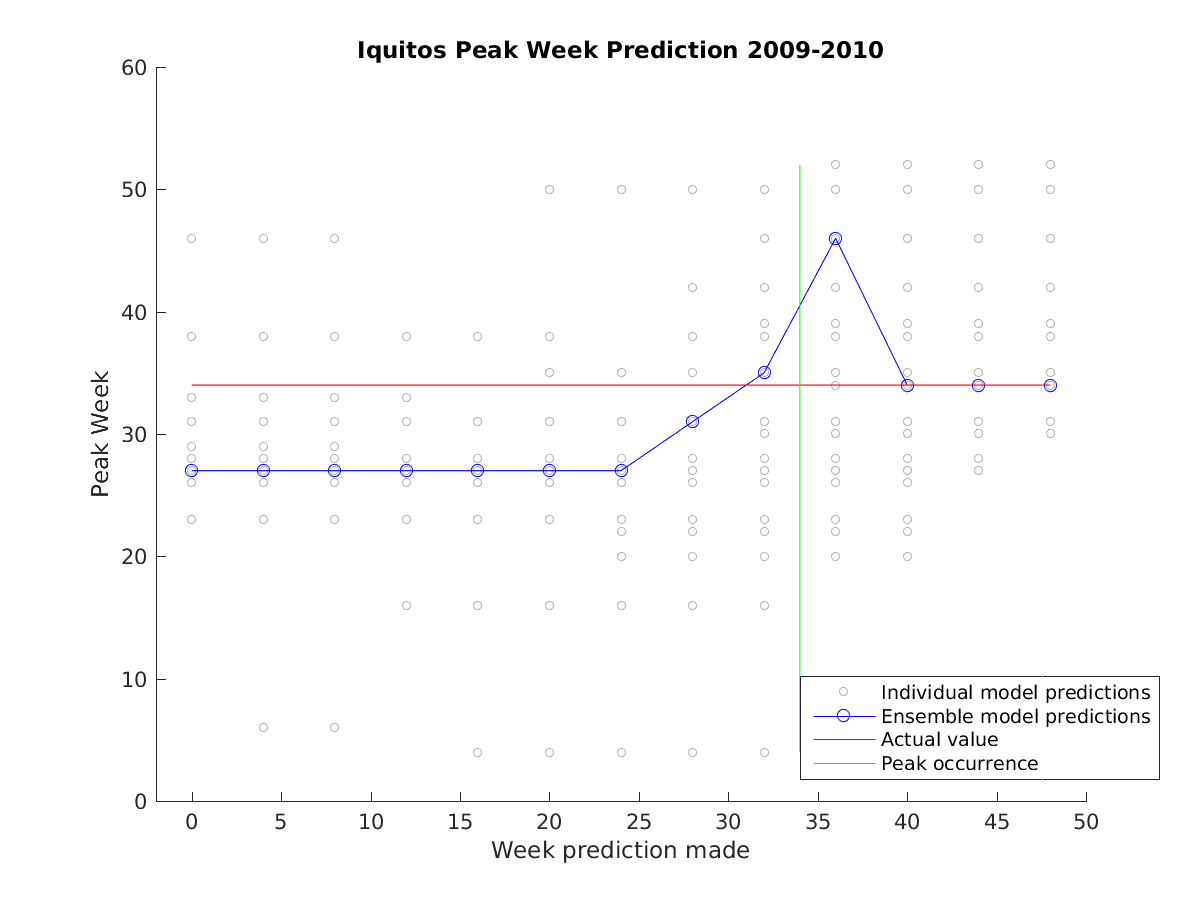

Supplement: S1 File — (ZIP) [file pone.0189988.s001.zip › S1_FigU.tif]

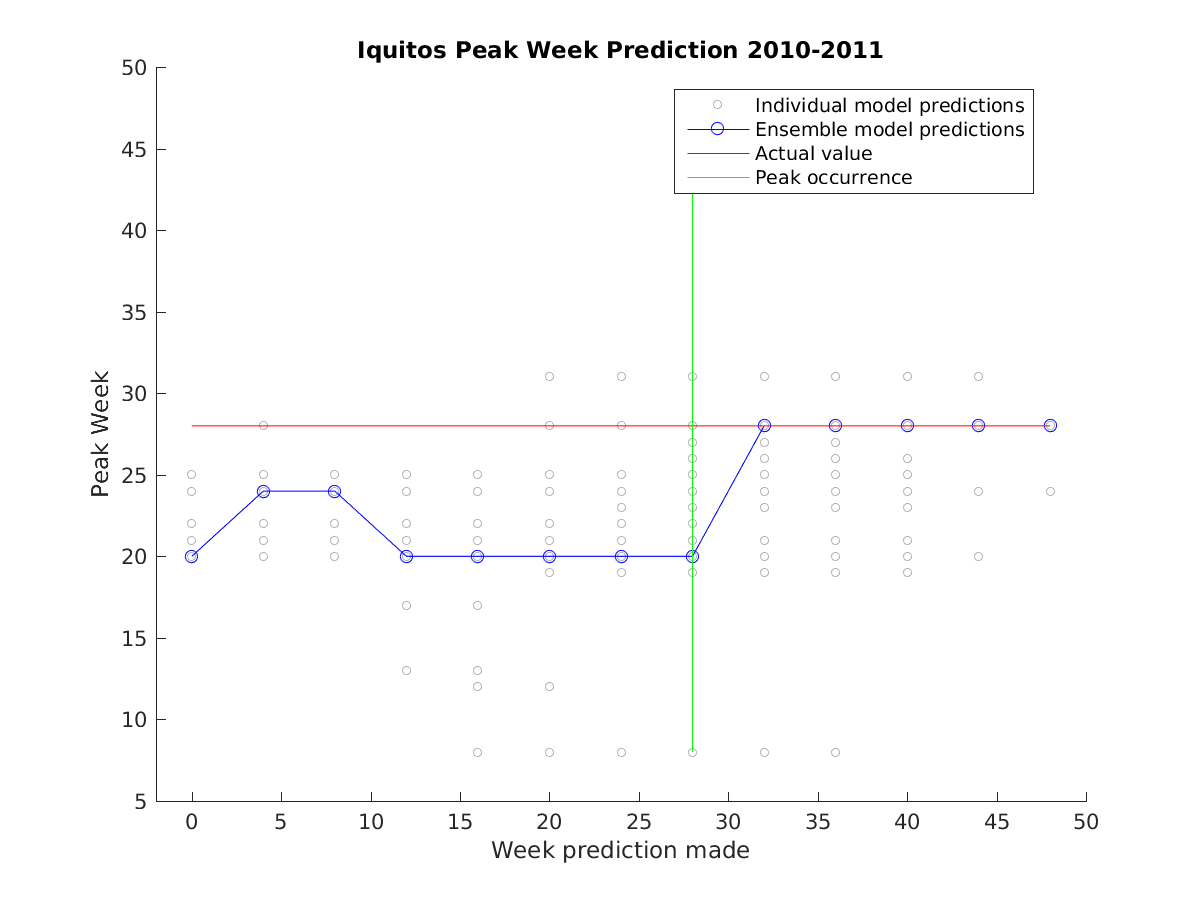

Supplement: S1 File — (ZIP) [file pone.0189988.s001.zip › S1_FigV.tif]

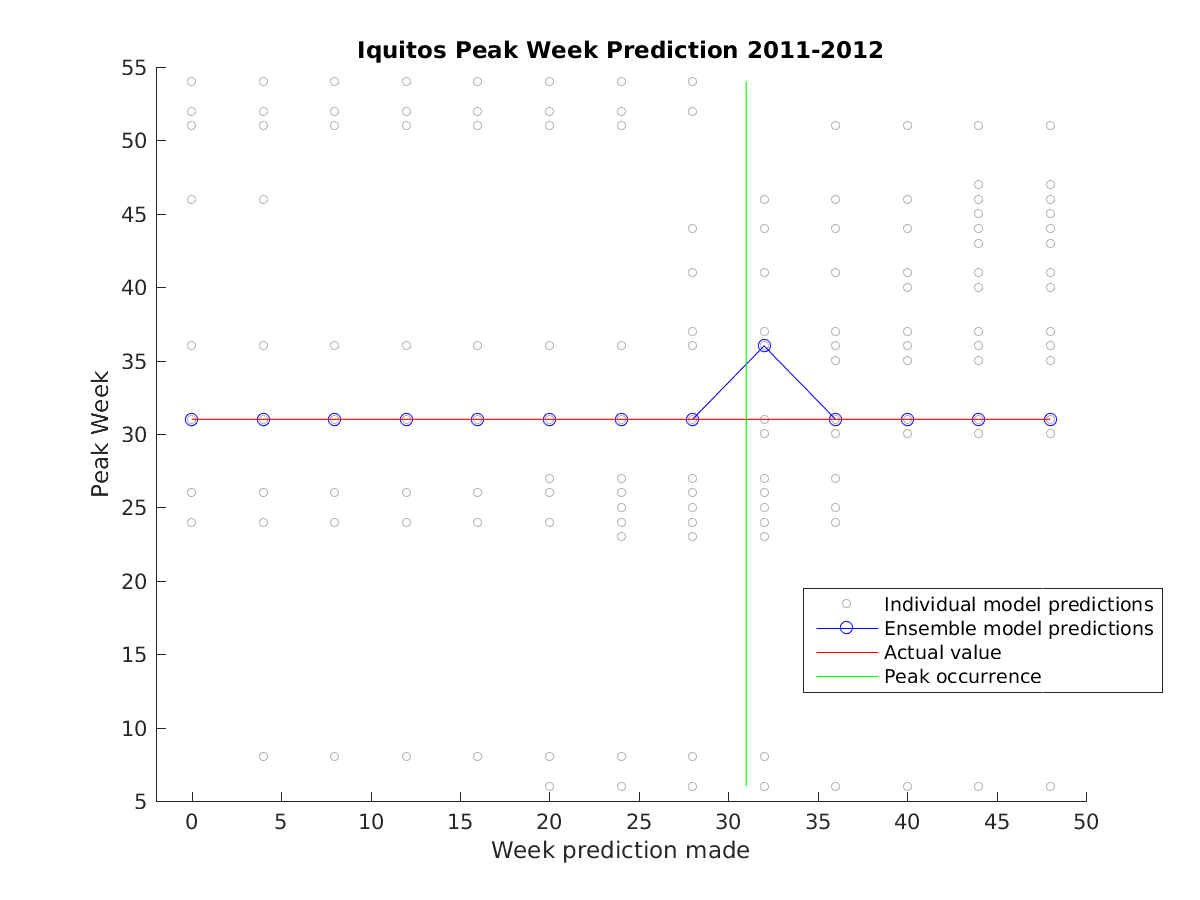

Supplement: S1 File — (ZIP) [file pone.0189988.s001.zip › S1_FigW.tif]

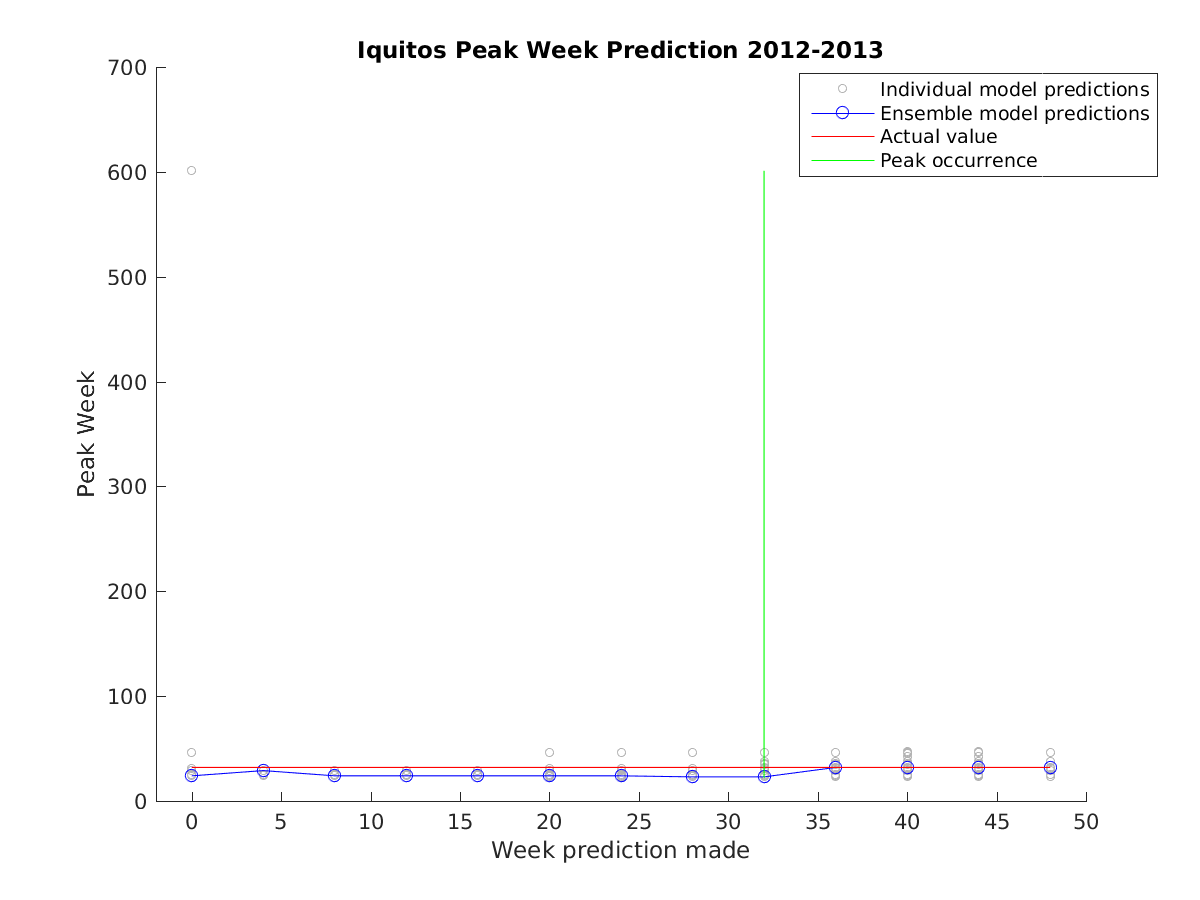

Supplement: S1 File — (ZIP) [file pone.0189988.s001.zip › S1_FigX.tif]

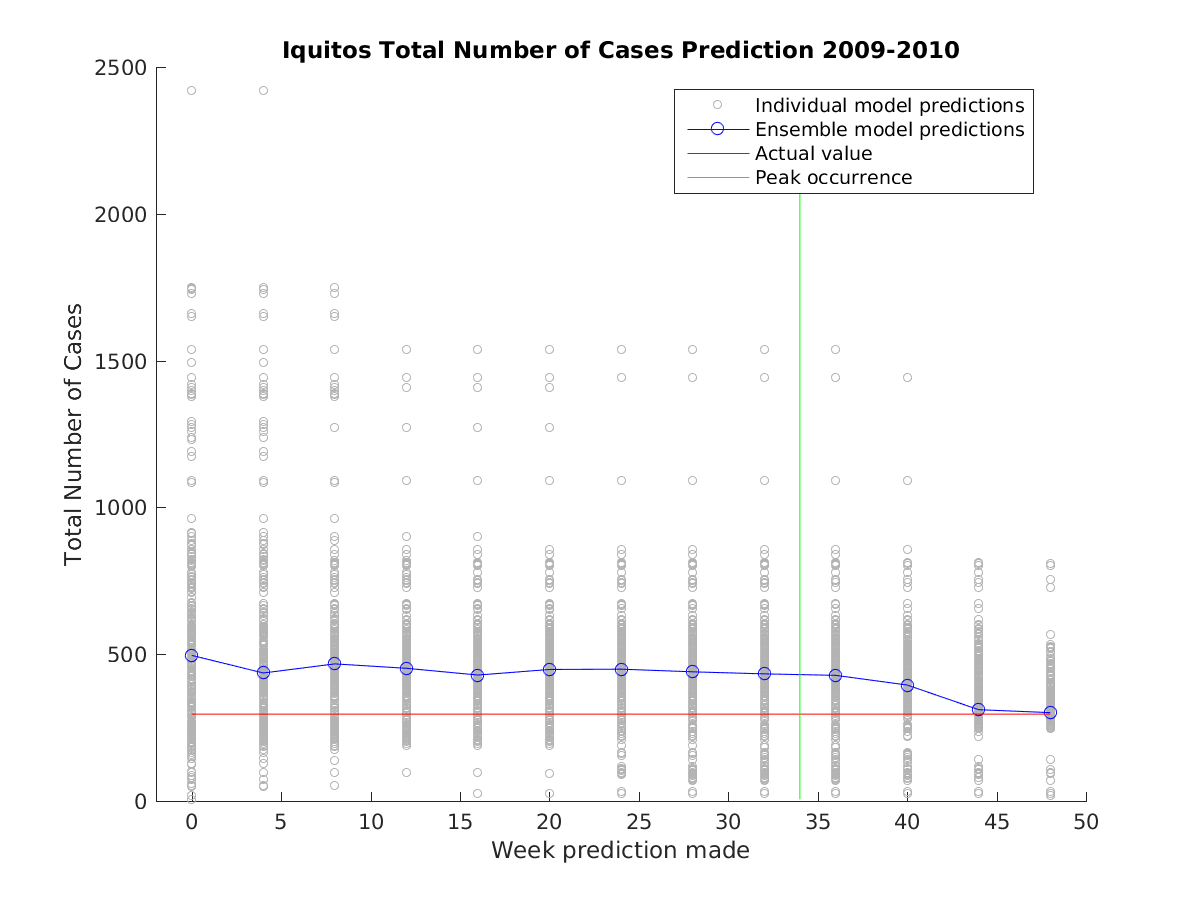

Supplement: S1 File — (ZIP) [file pone.0189988.s001.zip › S1_FigY.tif]

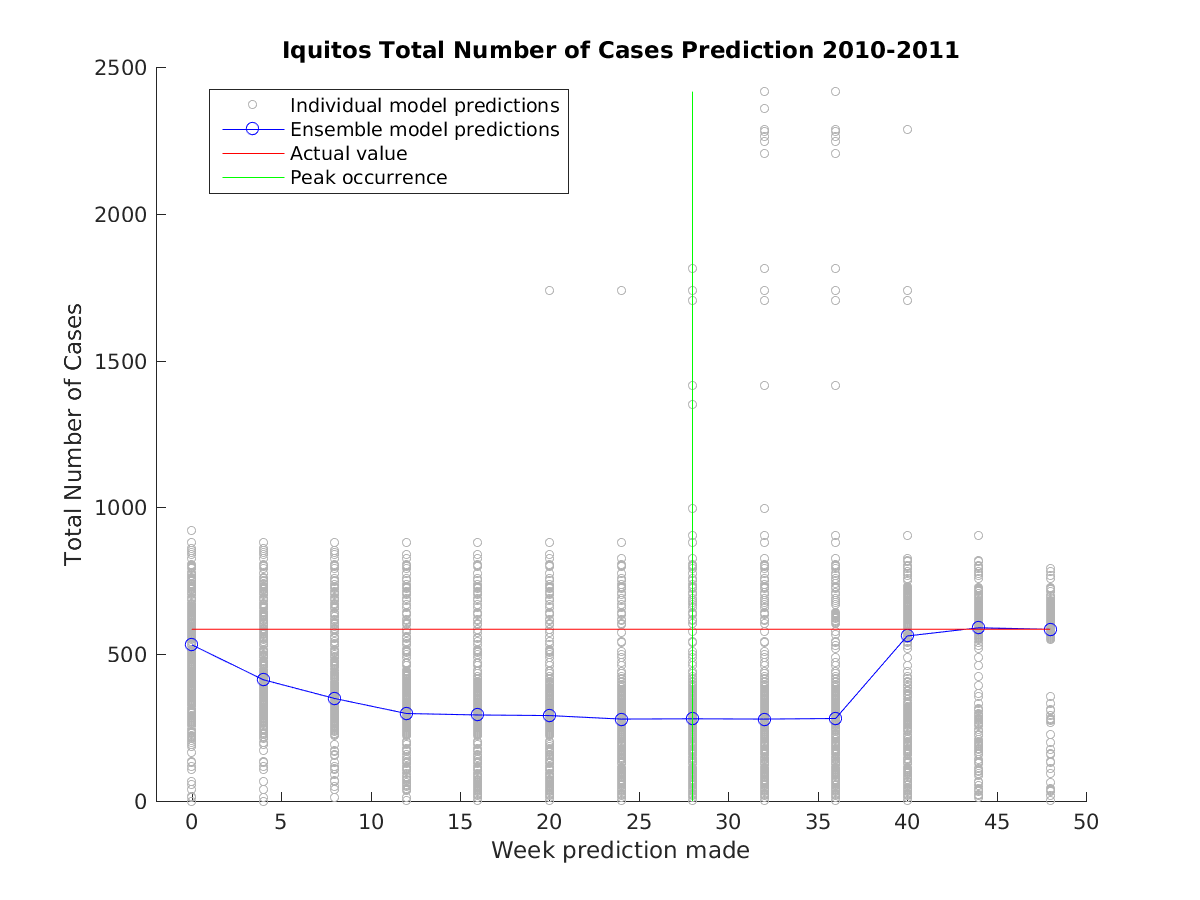

Supplement: S1 File — (ZIP) [file pone.0189988.s001.zip › S1_FigZ.tif]
